# Microgel‐Based 3D Bioprinting: A Convergent Strategy Integrating Material Design, Jamming Dynamics, and Biological Function

**DOI:** 10.1002/adhm.71319

**Published:** 2026-06-04

**Authors:** Elena Ghighină, Andreea Ioana Dinu, Jana Ghițman

**Affiliations:** ^1^ Advanced Polymer Materials Group National University of Science and Technology Politehnica Bucharest Bucharest Romania; ^2^ Center of Excellence in Bioengineering – eBio‐hub National University of Science and Technology Politehnica Bucharest Romania

**Keywords:** bio‐architectures, biomedical innovation, jamming effect, microgel‐based 3D matters, tissue‐like constructs

## Abstract

Driven by rapid technological innovation and an increasing demand for personalized medicine, microgel‐based bioprinting emerges as a versatile strategy for generating physiologically relevant tissue models and patient‐specific constructs. By integrating microgel systems with 3D bioprinting, this approach enables the creation of complex, cell‐laden architectures that closely recapitulate native tissue architecture and function. Pivotal to this potential are microgels that offer significant advantages over conventional hydrogel‐based bioinks, including tunable rheological behavior, enhanced print fidelity, and a supportive microenvironment for cell viability and maturation. This review outlines the fundamental principles and recent advances that shape this rapidly evolving field, focusing on microgel synthesis strategies and how their physicochemical design and rheological properties can be leveraged to precisely tailor their printability and biological performances. The role of microgels in 3D bioprinting is comprehensively discussed, focusing on their jamming behavior and ability to form tissue‐mimetic constructs. Emerging approaches for creating structurally and functionally advanced microgel‐based 3D matters are also explored, highlighting their transformative role in diverse biomedical applications. Finally, the review outlines key translational opportunities and challenges, emphasizing the promise of microgel‐based 3D constructs as a versatile platform for regenerative therapies and the development of clinically relevant tissues.

## Graphical abstract

Microgel‐based 3D printed constructs represent a compelling and versatile innovation for engineering architecturally complex, dynamically remodelable, and biocompatible structures with high structural fidelity and bioactivity. By integrating material design, biofabrication, and biological function, these systems enable the development of adaptive tissues, offering a conceptual framework for next‐generation, clinically relevant, and functionally integrated living systems.

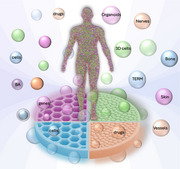

## 1 Introduction

Over the last two decades, the once distant worlds of materials science, bioengineering, and additive manufacturing have converged, transforming the landscape of biomedical innovation. This coalescence has not only redefined the design principles and fabrication of biomedical devices, but also the conceptualization of living systems, bridging the long‐standing gap between engineered materials and biological function [1, 2]. In this new framework, matter can be digitally guided to grow, adapt, and interact with its environment, opening possibilities for constructing tissues and organs that were once the domain of imagination [3, 4]. This advancement has turned 3D printing into a design language of living matter, through which cells, biomolecules, and smart materials can be orchestrated into complex, clinically relevant tissue‐like assemblies [3, 5, 6]. Once limited to inert polymers and metals, the field has rapidly evolved into the world of soft, living matter, guiding a new generation of bio‐fabrication in which form and function merge [7, 8].

**FIGURE 1:**
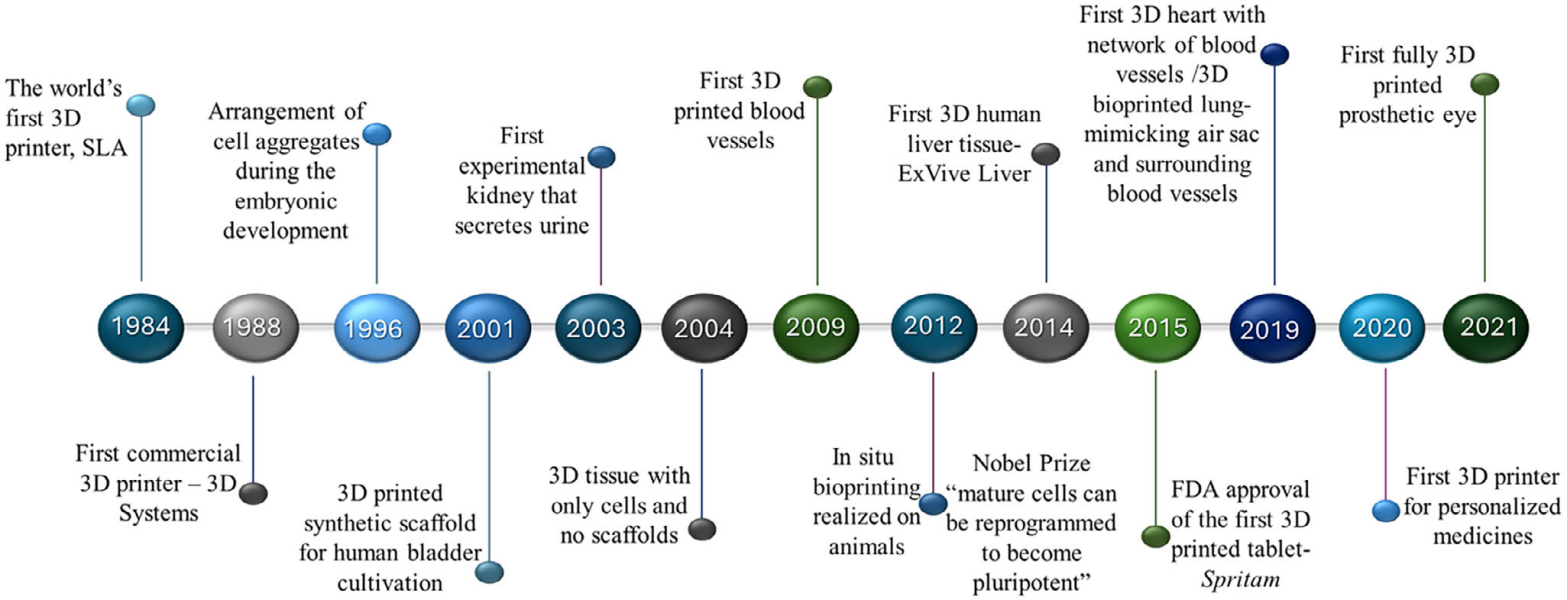
Timeline of some key milestones for 3D printed constructs.

Driven by this progress, the library of 3D printed constructs has advanced beyond purely structural or mechanical prototypes toward biologically oriented systems, designed to facilitate cell growth and tissue engineering (Figure 1) [4, 5, 9, 10, 11]. Reflecting this technological advancement, the U.S. Food and Drug Administration (FDA) has progressively recognized the clinical potential of 3D printing technologies, approving a range of medical devices from orthopedic implants to dental restorations, highlighting the growing confidence in their clinical translation [5].

Among the materials driving this transition, hydrogel‐based bioinks such as alginate, gelatin, and its methacrylated derivative gelatin methacryloyl (GelMA), hyaluronic acid played a central role in early bioprinting, owing to their highly hydrated network, tunable mechanics, and amenability in formulation along with intrinsic biocompatibility and biomimicry [12, 13, 14].

Albeit their promise, conventional bulk hydrogels face intrinsic limitations related to both printability and biological performances. Printability issues originate from the inadequate rheological behavior, low structural stability, specifically in complex multilayered constructs, and slow or uncontrolled crosslinking, whereas biological constraints are associated with cell‐damaging extrusion conditions, restricted nutrient transport, limited structural heterogeneity, and insufficient control over cell–material interactions (Table 1) [15, 16]. In light of these challenges, 3D constructs capable of actively responding to living cells remain mostly in the preclinical phase, requiring in‐depth investigations prior to clinical translation.

**TABLE 1:** Fundamental differences between conventional hydrogels and microgels in 3D bioprinting [6, 27, 28, 29, 30].

Features	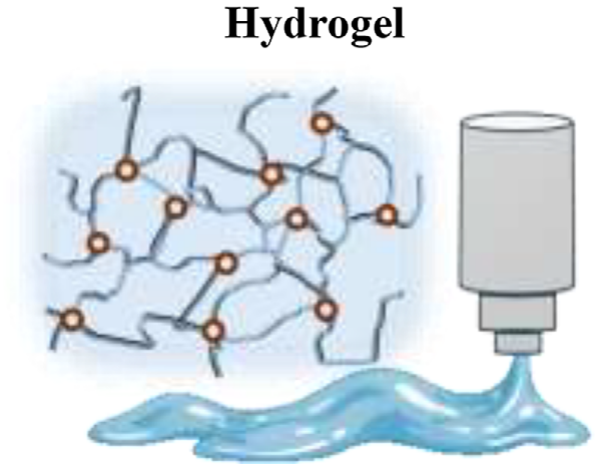	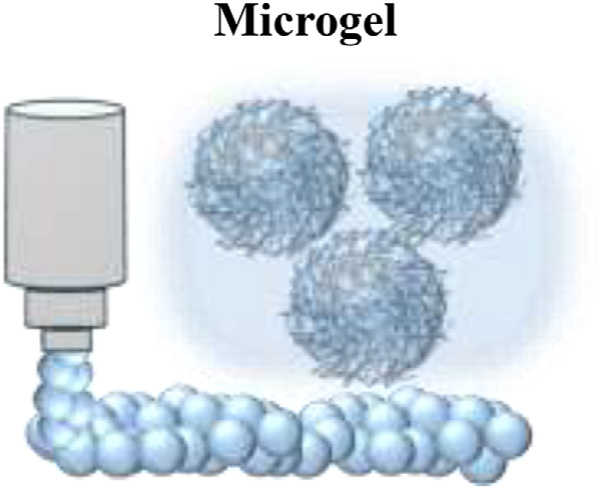
Architecture/structural organization	Continuous macromolecular 3D polymeric network, swollen in water.	Discrete swollen colloidal‐sized hydrogel particles (typically in the µm) suspended in a medium.
Surface area	Relatively low, determined by the outer surface of the block.	High surface‐to‐volume ratio enhances molecular exchange and cell interfacing.
Mechanical features	Soft and elastic macrostructures, with low tensile strength and modulated stiffness, depending on the composition and crosslinking density.	Softer and more deformable, highly tunable features, with high strength due to microporous structure and ability to interlock; High structural integrity.
Porosity and swelling behavior	Limited porosity (nanoporous structure); Swells uniformly when exposed to water or solvents, which may lead to mechanical weakening	Microporous structure allows better cell infiltration and nutrient diffusion. Swelling is restricted to individual particles, usually leading to faster response time.
Self‐healing & shape recovery	Low self‐healing ability; once fractured, recovery is difficult.	Good self‐healing ability owing to modular structure (microgel reassembly).
Injectability & Modularity	Requires invasive placement; less adaptable post‐formation.	Injectable, modular, and readily customizable for in situ applications.
Mass transport	Nutrient and waste diffusion are limited in larger constructions.	Enhanced mass transfer owing to interconnected porosity.
Cellular interactions	Basic cell encapsulation and ECM mimicry.	Microporous, cell‐permissive architecture suitable for cell infiltration, ECM deposition, and organized tissue formation.
Printing amenabilities	Low yield stress, poor shape fidelity, and a collapsed multilayered construct.	Sher‐thinning, high shape fidelity, stable complex multilayered construct.

Within this framework, microgel‐based 3D printed constructs have emerged as one of the most compelling and versatile innovations, offering new pathways to engineer complex, dynamic, and biocompatible structures that converge the microscale architecture and dynamic cell‐material interfaces, blurring the limits between synthetic materials and living tissues [7, 8, 17, 18, 19].

Microgels are micron‐scale hydrogel particles with highly tunable mechanical, chemical, and biological properties, representing a modular platform for creating soft, reconfigurable materials [20, 21, 22]. When densely packed, these particles form granular hydrogels, exhibiting solid‐like behavior at rest and fluid‐like properties under shear stress, a phenomenon known as the jamming transition [1, 23]. This unique rheological adaptability permits microgels to serve as self‐supporting bioinks, capable of flowing smoothly through printing nozzles while rapidly recovering structural integrity upon deposition, a property fundamental for high‐resolution and high‐fidelity 3D bioprinting [20, 22, 24]. By addressing intrinsic limitations related to structural fidelity and bioactivity of traditional hydrogel printing (Table 1), microgel platforms enable the fabrication of architecturally complex, dynamically remodelable, and functionally integrative living systems [8, 17]. Furthermore, precise tuning of microgel interfacial chemistry and the integration of bioactive signaling motifs are giving rise to smart, responsive bioinks capable of supporting cellular processes and adapting mechanically to their biological environment [25, 26].

1 **Box 1 | Microgel‐based bioinks: Structure, Function, Printability**

Microgel‐based bioinks are micron‐sized (10–500 µm) hydrogel particles that combine flowability with tunable mechanical and biological properties, enabling high‐fidelity 3D bioprinting of complex tissues [24, 26]. These discrete architectures allow independent control over stiffness, porosity, and degradation, closely mimicking native extracellular matrix [27, 28].

Advantages:

**High printability**: Shear‐thinning behavior and rapid recovery maintain structural precision [28].

**Cell‐friendly**: Microgels provide a protective microenvironment, supporting survival and function.

**Enhanced transport**: Interstitial pores improve nutrient and oxygen diffusion [24].

**Customizable biofunctionality**: Surface chemistry and cargo (growth factors, ECM motifs, drugs) can be tailored for specific tissues [13, 29].

By merging structural tunability with biological compatibility, microgel bioinks represent a promising and versatile platform for next‐generation tissue engineering and regenerative medicine.

Beyond the laboratory, the commercial success of microgel systems for biomanufacturing underscores their broad applicability and translational potential, reflecting the practical significance of jamming‐inspired materials in contemporary biomedicine [31]. Bioprinting companies and biomaterials suppliers now offer ready‐to‐use microgel inks and modular bioinks based on alginate, gelatine methacryloyl (GelMA), polyethylene glycol (PEG), and other biopolymers [31]. These products have found applications in tissue engineering, regenerative medicine, and drug delivery, demonstrating that microgel‐based materials can provide both mechanical support and biological functionality [17, 32]. At the same time, the clinical market for 3D printed biomaterials continues to expand, driven by the demand for patient‐specific solutions, personalized therapies, and minimally invasive biofabrication technologies [3, 4, 33].

In this respect, the concept of microgel‐based 3D printed matters represents not only a technological innovation but also a conceptual framework for next‐generation biofabrication, integrating injectability, porosity, shear‐thinning behavior, and biochemical tunability into a single, highly versatile platform [21, 32]. These features endow the materials with both engineering precision and biological relevance, supporting cell encapsulation, nutrient exchange, and tissue maturation in architectures that mirror the native extracellular matrix (ECM) [1, 7, 23].

Collectively, these innovations establish a foundation for functionally integrated constructs designed to guide tissue repair, regeneration, and organ‐level modeling [4, 32]. By disentangling the complex interplay between materials design, cell biology, and additive manufacturing, researchers are leveraging microgels to create constructs that are mechanically robust, biologically functional, and potentially clinically translatable.

Reflecting the growing interest and rapid advancement of this field, several review articles explored the emergence of microgel‐based systems for biomedical and biofabrication applications, primarily focusing on their synthesis strategies and physicochemical properties, emphasizing their advantages over traditional bulk hydrogels in improving printability and biological performances [25, 34, 35]. Subsequent works have further analyzed granular hydrogel bioinks and microgel‐based systems for 3D bioprinting, introducing their role as modular building blocks and highlighting the rheological behavior and potential as printable biomaterials for engineering complex tissue constructs [1, 7, 21, 24]. Albeit these studies have significantly contributed to the field, most of them address individual aspects of microgel systems without fully integrating the relationships between microgel design, jamming behavior, printing performance, and biological outcomes, which collectively influence their functional performance and translational potential.

Building on these advances, the present review explores the emerging landscape of microgel‐based 3D printed matters, providing an integrated perspective linking microgel physicochemical design, jamming behavior, and 3D printing and bio‐fabrication performances with biological functionality in engineered tissues. Besides summarizing synthesis strategies and biofabrication approaches, it discusses how the interplay between microgel architecture, rheological properties, and processing parameters influences structural fidelity, cell behavior, and tissue development. In addition, it addresses the practical, scientific, and regulatory challenges that remain critical barriers to the clinical translation of microgel‐based 3D constructs but are often relatively overlooked in existing reviews. By bridging material design, biofabrication strategies, and biological outcomes, this review aims to afford a conceptual framework for advancing microgel‐based 3D matters toward next‐generation bio‐fabricated tissues that are dynamic, programmable, intrinsically alive, and clinically relevant.

## 2 Microgel Fabrication Strategies: Building Blocks for Complex Bio‐Architectures

From droplet to structure, the art of microgel formulation allows the creation of highly controlled bio‐architecture, unlocking the potential of 3D printing in various fields of biomedicine. Precise control over microgel size, shape, and functional properties enables these tiny building blocks to be assembled into advanced, multifarious structures that closely mimic natural tissue environments [36]. By tailoring their composition and responsiveness, microgels serve not only as structural scaffolds but also as vehicles for controlled drug delivery [37, 38], signaling [22, 39], or cell encapsulation [40, 41]. Therefore, careful design and formulation of microgels represent the pillar for constructing advanced 3D‐printed sophisticated bio‐architectures and functional tissues with precisely tailored features for targeted biomedical applications [22].

To date, a broad library of microgel fabrication strategies has been developed, which, depending on material properties and target applications, can be generally classified into several categories: batch emulsification, membrane emulsification, microfluidics, electrohydrodynamic spraying (EHDS), wet‐spinning, lithography, precipitation polymerization, and mechanical fragmentation.

Understanding the synthesis and formulation of microgels is primordial, as the chosen methods directly impact their architecture, functionality, and suitability for subsequent applications.

Accordingly, the growing interest in these versatile materials is reflected in the increasing number of review articles approaching microgel fabrication and biofabrication strategies. Several reports are focused on the formulation of microgel‐based bioinks and the processing during extrusion‐based bioprinting and post‐printing stages [7, 25]. In a comprehensive review on microparticulate hydrogels for biomedical applications, Daly et al. [21] provided a comprehensive overview of the commonly used microgel fabrication strategies, while other techniques associated with specific operational constraints, such as precipitation polymerization, membrane emulsification, and wet spinning, are beyond the scope of the review. Similarly, Xie et al. [35] described microgel fabrication approaches using an alternative classification such as bulk crushing, multiphase emulsion, auxiliary dripping, and lithography, while Feng et al. [32] summarized the main strategies for microgel formulation, primarily underlying the assembly mechanisms, including chemical reactions, physical interactions, cell–cell interactions, and external driving forces, rather than providing a detailed comparison of fabrication approaches and their processing parameters.

Building on these reports, the following section reviews the key strategies for microgel fabrication, highlighting their principles, advantages, and limitations (Table 2) in creating versatile building blocks for advanced 3D printed bio‐architectures. By connecting microgel synthesis approaches with their rheological properties and printing outcomes, this section highlights how fabrication parameters influence not only particle architecture but also printability, structural fidelity, and biological functionality in engineered tissues.

**TABLE 2:** Performances and drawbacks of microgel fabrication strategies.

Methods	Benefits	Limitations	Particle Geometry	Types of Polymers Used	Additional Information/key factors	Ref.
Batch‐ Emulsion	High throughput, versatile, flexible, multifarious polymer combinations	Low control over size distribution, limited to batch processes	Spherical, irregular	PAM, Gelatine, Alginate, PVA	Widely used for bulk microgel manufacturing, particularly in drug‐delivery emulsions, but precise control of size and uniformity remains challenging.	[42, 43]
Mechanical Fragmentation	Simple and economical, versatile across polymer systems, high scalability	Poor control over particle size, difficult to achieve uniformity in shapes	Irregular, spherical, fragmented	PAM, PVA Polystyrene, PEG‐based hydrogels	Enables large‐scale, cost‐effective production of diverse polymer‐based microgels, though control over particle size and uniformity can be limited.	[44, 45]
Membrane Emulsification	Produces uniform microgels, high throughput, industrial scalability	Restricted to certain polymers, demands precise flow control	Spherical	Alginate, Gelatine, PAM, PEG‐based polymers	Commonly applied in drug delivery and tissue engineering for its size uniformity and scalability. The efficiency depends on optimizing the flow and membrane parameters.	[46, 47]
Precipitation Polymerization	Simple and scalable, produces highly crosslinked microgels, ideal for drug delivery applications	Poor control over morphology requires careful solvent selection	Spherical	PNIPAM, PEG‐based hydrogels, Polystyrene, Acrylic copolymers	Well‐suited for large‐scale production due to its simplicity, though particle size control can be challenging, especially useful for stimuli‐responsive microgels.	[48, 49]
Electrohydrodynamic‐spraying	Generates ultra‐fine (nanosized) microgels, compatible with biomaterials, suitable for drug delivery applications	Requires a high‐voltage complex setup, limited scalability	Spherical, irregular	Alginate, Chitosan, PCL, GelMA, PLGA	The method is preferred for drug delivery systems requiring high porosity or controlled release, while scale‐up is challenging due to high‐voltage requirements and complex equipment.	[50, 51]
Wet‐spinning	High control of microgel geometry generates high‐aspect ratio microfibers. Crosslinking versatility requires no specialized equipment	Restricted material range, Mechanical fragility, Limited geometric complexity, Process sensitivity	Cylindrical, ribbon‐, rod‐shaped, core–shell, hollow	Alginate, Hyaluronic acid, Gelatin/GelMA, Chitosan, PEG, PCL, Pluronic derivatives	Versatile platform for producing anisotropic hydrogel microstructures with controlled geometry and cell‐instructive properties, suited for tissue engineering and biofabrication applications.	[52, 53]
Microfluidics	Precise size and shape control, Produces adaptive microgels, Proper for biomedical applications	Low throughput, High equipment cost, technically demanding	Spherical, core–shell, Janus, anisotropic	Alginate, Gelatine, Hyaluronic acid, Chitosan, PNIPAM, PEG	Ideal for producing microgels with tailored functional properties, such as drug encapsulation or imaging, as microfluidic devices provide precise control over droplet formation.	[54, 55]
Lithography	High‐resolution microparticle patterning supports complex architectures, Applicable to biosensors and tissue scaffolds	Material‐limited, Facility‐dependent, Low throughput, Cell delivery constraints, Cost‐inefficient at scale	Custom‐tailored, anisotropic	PEGDA, GelMA, Polyacrylates	Primarily employed in high‐precision applications such as biosensors and diagnostics, with photolithography enabling precise, tailored microgel structures, though it requires costly equipment.	[56, 57]

*Abbreviation*: PAM—Poly(acrylamide), PVA‐ Polyvinyl alcohol, PEG—Polyethylene glycol, PNIPAM—Poly(N‐isopropylacrylamide), GelMA ‐Gelatine methacryloyl, PCL—Polycaprolactone, PLGA—Poly(lactic‐co‐glycolic acid, PEGDA ‐Polyethylene glycol diacrylate.

### 2.1 Batch‐Emulsion

**FIGURE 2:**
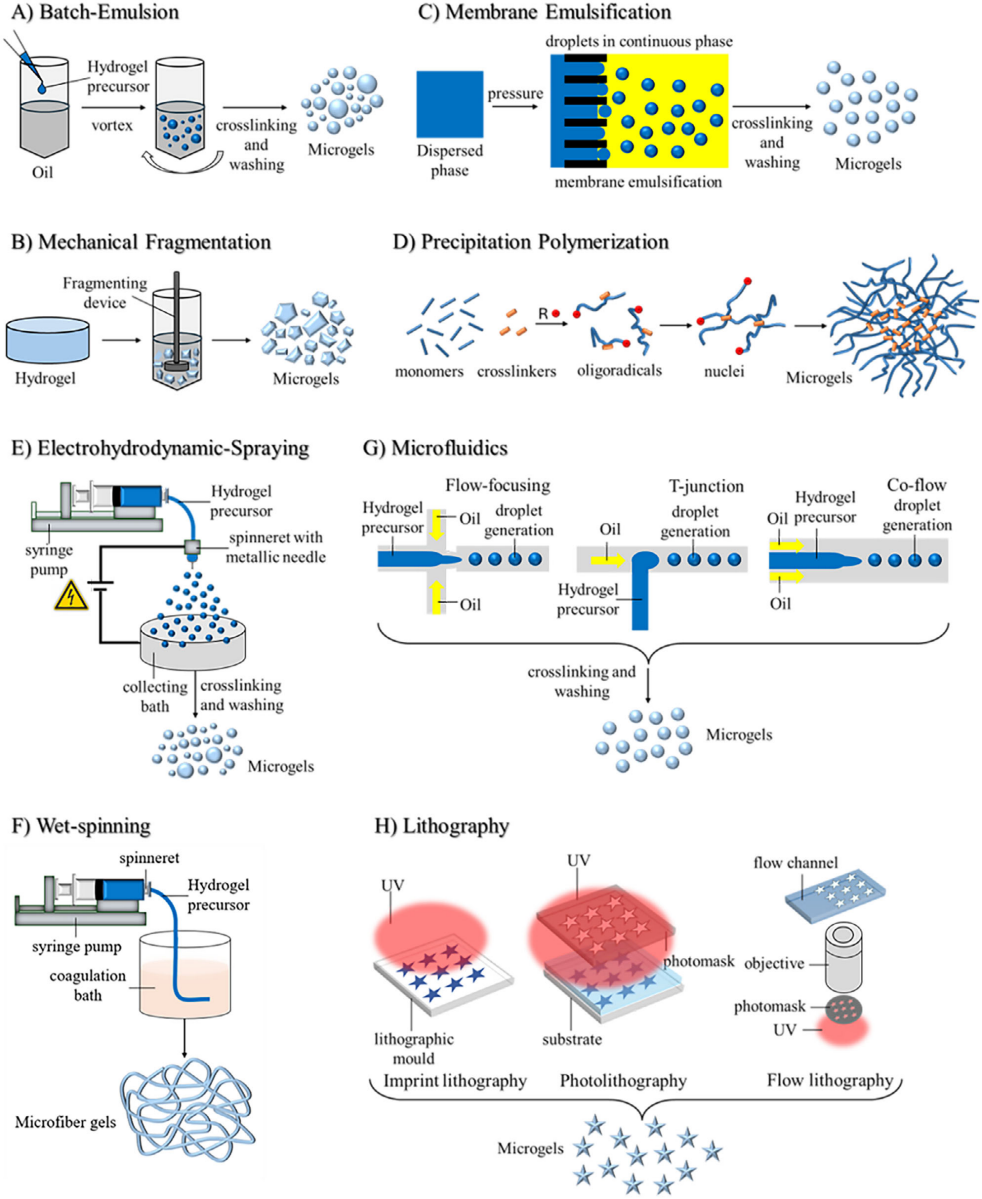
Strategies for engineering microgels. (A) batch‐emulsion: immiscible liquids are mixed in bulk to produce droplets, which are then crosslinked to create microgels; (B) mechanical fragmentation: preformed hydrogels are broken down into smaller particles using mechanical energy; (C) membrane emulsification: a dispersed phase is forced through membrane pores into an immiscible continuous phase, generating emulsion droplets that are crosslinked into microgels; (D) precipitation polymerization: monomers, crosslinkers, and initiators are initially soluble in the reaction medium, but as polymerization progresses, growing polymer chains reach a critical size, become insoluble, and precipitate, forming microgels; (E) electrohydrodynamic‐spraying: electrical forces are applied to charged liquid streams to form droplets that are subsequently crosslinked into microgels; (F) wet‐spinning: a polymer or pre‐gel precursor solution is extruded through a spinneret into a coagulation bath, where solidification occurs, producing high‐aspect ratio microfiber gels; (G) microfluidics: droplets generated via flow‐focusing, T‐, or co‐flow junctions are crosslinked to form uniform microgels; (H) lithography: masks or molds act as microscale templates to precisely shape and define microgel structure.

Emulsion represents a fundamental strategy for generating microgels, in which one liquid is dispersed at microscopic droplets within another immiscible liquid under the influence of interfacial tension [21]. In typical emulsion‐based processes, aqueous polymer precursors (monomers, initiators, and crosslinkers) are emulsified in an oil phase by mechanical stirring, forming water‐in‐oil droplets (Figure 2A and Table 2). These are subsequently solidified by photocrosslinking, thermal curing (e.g., radical polymerization in acrylated or methacrylated systems) [42] or temperature‐induced gelation of thermosensitive polymers [58]. Surfactants are commonly added to stabilize the emulsion. Batch emulsions enable high‐throughput production of spherical droplets ranging from 1–10 µm up to several hundred microns. However, droplet size and dispersity depend strongly on stirring conditions [43], often leading to polydisperse populations and batch‐to‐batch variability. This heterogeneity can affect diffusion dynamics and drug release profiles, determining a more variable and pronounced initial burst release compared to microfluidic approaches [59]. Despite these limitations, batch‐emulsions offer key advantages: operational simplicity, scalability, high particle yield, and compatibility with biologics, including small molecules, growth factors, and cells, with viabilities exceeding 80% [42, 58]. Nonetheless, when carefully optimized and executed with a clear understanding of formulation parameters, these limitations become largely manageable, and methods remain highly versatile and scalable, even if they enable limited control over particle size distribution and uniformity.

### 2.2 Mechanical Fragmentation

Mechanical fragmentation produces microgels by physically breaking down bulk hydrogels into irregularly shaped microscale particles (Figure 2B and Table 2). In this approach, a preformed, crosslinked hydrogel is subjected to compressive or shear stress, with particle size influenced by processing parameters. For instance, extrusion through steel meshes can produce microgel fragments with mesh‐dependent sizes; sequential extrusion and sieving produced injectable zwitterionic microgels (15–30 µm) with improved size uniformity [44]. Also, gelatine microgels with uniform size and irregular morphology have been obtained by low‐temperature gelation followed by crushing and extrusion [60], whereas agarose hydrogels have been disintegrated through blending, yielding particles of approximately 100 µm at higher polymer concentrations and gradually smaller fragments at lower concentrations [45]. The main benefits of mechanical fragmentation are simplicity and throughput production in a single workflow. However, because of the limited control over particle size and morphology, this method is more appropriate for applications where precise uniformity is not critical. Moreover, its compatibility with viable cell encapsulation remains uncertain, as this aspect has not yet been systematically investigated.

### 2.3 Membrane Emulsification

Membrane emulsification enables the production of size‐controlled droplets that can subsequently be crosslinked into monodisperse microgels widely used in drug delivery, tissue engineering, and biosensing [61, 62]. In this method, a dispersed polymer solution is pressed through a porous membrane into an immiscible continuous phase. Uniform droplets form at the membrane surface and detach under shear stress, then gel to yield microgels [63] (Figure 2C and Table 2). Droplet size is primarily governed by membrane pore size, applied pressure, shear rate, and phase viscosities, while pore geometry influences uniformity [46]. The dispersed phase generally consists of natural or synthetic polymers (e.g., alginate, gelatine, hyaluronic acid, chitosan, PEG derivatives), while the continuous phase may form simple (W/O, O/W) or multiple (O/W/O, W/O/W) emulsions, which are stabilized by a surfactant. The obtained droplets are crosslinked (ionically, thermally, chemically, or photochemically) and purified by centrifugation or filtration.

Formulation parameters (e.g., polymer and surfactant concentration, emulsification rate, water/oil ratio, oil viscosity) further dictate size and morphology: higher polymer concentrations produce spherical microgels, while lower concentrations yield irregular shapes; increased surfactant levels usually reduce droplet size [64]. The method supports cell encapsulation despite potential oil‐ or surfactant‐related cytotoxicity, is easy to handle, requires no complex equipment, and provides high yield and energy efficiency, making it scalable for industrial applications. For instance, Lipke et al. [65] generated cancer cell‐laden microgels to create tumor spheroid models for drug screening, while Tian et al. [66] fabricated alginate microgels containing mesenchymal stem cells and magnetic nanoparticles, assembling them into magnetically actuated microrobots for targeted delivery. Maleki et al. [47] further demonstrated the scalability for producing 10–100 µm microcapsules via interfacial complexation and controlled shell formation kinetics. However, maintaining accurate monodispersity and batch‐to‐batch consistency remains challenging, while size heterogeneity can affect encapsulated cell number or drug dose uniformity.

### 2.4 Precipitation Polymerization

Precipitation polymerization is a widely used approach for synthesizing highly crosslinked, monodisperse microgels, valued for its simplicity and surfactant‐free operation. In this process, monomers, crosslinkers, and initiators are initially soluble in the reaction medium (typically water or water/organic mixtures). As polymerization proceeds, growing polymer chains reach a critical length, become insoluble, and nucleate into polymer‐rich microgel particles (Figure 2D and Table 2). Maintaining low monomer concentrations is critical to favor controlled particle formation and avoid bulk gelation. Common monomers include N‐isopropylacrylamide (NIPAm), acrylamide (AAm), acrylic acid (AAc), and methacrylic acid (MAAc). NIPAm is particularly important for its lower critical solution temperature (LCST) near 32°C, which imparts thermoresponsive behavior [67]. A bifunctional crosslinker, typically N,N′‐methylenebisacrylamide (BIS), establishes the 3D network, while ammonium persulfate (APS) initiates free‐radical polymerization at 60°C–80°C [68]. Caputo et al. [48] demonstrated that chitosan‐based microgels prepared via free‐radical precipitation polymerization can be finely tuned by adjusting co‐monomer feed ratios (AAc, AAm, NIPAm, NIPMAm), using BIS as crosslinker and APS as initiator. By modulating composition, they achieved monodisperse particles with controllable swelling and dual pH‐ and temperature‐responsiveness. Similarly, Khan et al. [49] reported chitosan–poly(MAAc) microgels with feed‐dependent physicochemical properties, including volume phase transitions, colloidal stability, electrical behavior, and dynamic swelling. Precipitation polymerization can also generate core–shell architectures: cores serve as seeds for shell growth, producing microgels with interpenetrating networks. For example, DNA–poly(NIPMAm) microgels showed preserved core integrity and size‐ and hydrophobicity‐dependent accessibility, highlighting the potential for tunable functional microgels in biomedical applications [69]. The primary advantages of this method lie in operational simplicity under aqueous conditions and versatility in functional monomer incorporation. However, successful particle formation requires polymer chains to become insoluble at the reaction temperature, limiting the use of highly hydrophilic monomers. As a result, co‐monomer content is constrained by hydrophilicity, e.g., acrylic acid can be copolymerized into pNIPAm microgels at 15%–20%, whereas primary amine monomers are typically restricted to only a few percent [70].

### 2.5 Electrohydrodynamic Spraying

Electrohydrodynamic‐spraying (EHDS) employs high‐voltage electric fields to atomize hydrogel precursors into microscale droplets, enabling the fabrication of 3D microgels, including high aspect ratio structures, for drug and cell delivery applications. In this process, the polymer solution is extruded through a needle connected to an electrode; electrostatic forces overcome surface tension at the tip, generating droplets that are collected in a crosslinking bath (Figure 2E and Table 2). Alginate and chitosan are usually used owing to their ionic or electrostatic crosslinking [71], while photocrosslinkable polymers can be solidified in light‐irradiated baths. Microgel size, shape, and morphology depend on both ambient and technical parameters [72], but the dispersity is often >5% [50]; stronger electric fields improve size reduction but may compromise cell viability. The monodispersity of microgels used in diverse areas of biomedicine can be improved by employing alternating vs. direct current and filtration strategies [73, 74]. EHDS‐produced microgels are highly effective for cell encapsulation, as they avoid the use of oils or surfactants. For example, Wang et al. [75] encapsulated natural killer cells in sodium alginate (SA) porous microgels, protecting the cells while enabling sustained release of perforin and granzymes for potent antitumor activity. Also, Song et al. [73] encapsulated primary human pancreatic cancer cells in SA/carboxymethyl cellulose (CMC) microgels, producing highly uniform 3D tumor spheroids that maintained cell viability and proliferative capacity. This technique allows the generation of porous, core–shell, and structurally complex microgels that support cell viability and controlled therapeutic release, underlining its versatility for cancer therapy, tissue regeneration, and neural engineering applications [51, 76].

### 2.6 Wet‐Spinning

Wet‐spinning is a controllable technique for producing high‐aspect ratio microfiber gels, in which a polymer or pre‐gel precursor solution is extruded through a spinneret into a coagulation or crosslinking bath, where solidification occurs (Figure 2F and Table 2). In hydrogel systems, precise control over flow dynamics, spinneret geometry, and in situ gelation kinetics enables the fabrication of anisotropic microgels with fiber‐ [77], ribbon‐ [52], or rod‐like [53] morphologies. Microgel architecture is governed by key processing parameters, including spinneret design, flow‐rate ratios (in coaxial systems), diffusion‐controlled crosslinking, and precursor solution rheology [38]. Circular spinnerets generate continuous cylindrical fibers, whereas slit geometries yield flattened ribbons; rod‐like microgels can be obtained via controlled filament breakup, such as hydrodynamic instabilities, segmented flows, or post‐spinning fragmentation. For example, Lee et al. [52] reported the development of anisotropic microribbons (µRBs) as granular bioinks, which showed shear‐induced alignment during extrusion, guiding mesenchymal stromal and endothelial cell organization, with increased µRB stiffness enhancing further cellular alignment and accelerating osteogenic differentiation. The system was also applied to model breast cancer–bone metastasis, enabling spatial patterning of multiple cell types and replicating invasion at tissue interfaces. Ma et al. [78] introduced an improved wet‐spinning approach to produce microfiber gels without post‐treatment steps, using sodium alginate (SA), methacrylated hyaluronic acid (MeHA), and the photo‐initiator LAP. Extrusion into a calcium chloride (CaCl_2_) coagulation bath induced rapid ionic crosslinking of SA, generating partially stabilized microfiber gels without post‐treatment. Further photo‐crosslinking facilitated direct assembly into microporous constructs, supporting applications such as articular cartilage regeneration.

### 2.7 Microfluidics

**Microfluidics** is a relatively recent technique that allows the controlled generation of uniform droplets by injecting a dispersed phase into a continuous immiscible phase, where interfacial shear forces and surface tension drive the droplet formation (Figure 2G and Table 2) [79]. Among microfluidic approaches, flow‐focusing designs are the most versatile, while T‐junction and co‐flow geometries are also widely used. Droplet size (typically 5–500 µm) and morphology are precisely determined by channel architecture and flow‐rate ratios, allowing exceptional control over microgel dimensions and structure [54]. Successful fabrication requires low‐viscosity precursor solutions for smooth flow and rapid crosslinking to prevent droplet coalescence. Crosslinking may occur on‐ or off‐chip, with on‐chip strategies preferred for improved stability. Photopolymerization of macromers such as PEGDA and GelMA is popular, allowing rapid gelation under light exposure [80], while thiol‐ene chemistries and thermally induced gelation (e.g., agarose, gellan gum) provide alternative routes. These capabilities make microfluidics particularly well‐suited for producing monodisperse, functional, and high aspect ratio microgels for applications in drug delivery and diagnostics [55]. A major strength of microfluidics is its ability to encapsulate small molecules, proteins, and cells with high precision. Monodispersity ensures predictable release kinetics and controlled cell loading, while cell‐laden microgels provide supportive 3D microenvironments that maintain long‐term viability and proliferation.

For example, Utech et al. [81] fabricated monodisperse sodium alginate (SA) microgels (10–50 µm) via droplet microfluidics, in which mesenchymal stem cells maintained high viability and stable proliferation over 15 days. Recent advances have combined microfluidics with emulsification to produce microgels with complex architectures beyond simple core–shell geometries [82], while commercial devices now allow the generation of uniform hollow microgels [83]. Nonetheless, reliance on oils and surfactants can raise cytotoxic risks for sensitive biomolecules or cells, driving interest in biocompatible aqueous alternatives. Although microfluidics offers unique control over particle uniformity, its principal limitation is low throughput, particularly for small droplets. This constraint can be partially mitigated and the scalability improved by using parallel multi‐junction devices.

### 2.8 Lithography

Lithographic techniques fabricate microgels by spatially controlled photopolymerization of hydrogel precursors at the microscale, offering exceptional precision over particle size, geometry, and architecture. Typically, a photoinitiator‐containing precursor is patterned using UV light through masks or molds, such as PDMS templates (Figure 2H and Table 2) [84]. The main approaches include imprint lithography, photolithography, flow lithography, and stop‐flow lithography.

Imprint lithography relies on filling patterned molds followed by curing, although limited precursor fluidity can compromise structural fidelity; vacuum‐assisted micromolding improves resolution by eliminating trapped air [56]. Photolithography instead uses patterned UV exposure to selectively crosslink defined regions, enabling accurate control of microgel dimensions. Acrylated PEG systems are widely applied owing to their tunability, cytocompatibility, and rapid radical polymerization, while chemically modified natural polymers (e.g., hyaluronic acid, gelatine) and thiol‐ene chemistries expand material versatility. However, conventional photolithography is generally limited to relatively simple geometries unless multiphoton systems are used. Flow lithography extends this concept by polymerizing sections of a continuously flowing precursor, increasing throughput and enabling compositional heterogeneity through co‐flows [85]. Compared to conventional microfluidics, flow lithography alleviates cell viability issues associated with narrow microchannels. To enhance biocompatibility, cells can be attached post‐synthesis, often requiring functional linkers and multistep conjugation.

Stop‐flow lithography (SFL) further simplifies the process: the precursor flow is paused, exposed to patterned light to form anisotropic microgels with complex geometries and high aspect ratios, and then resumed to release the solidified particles. This method provides tight control over size, shape, and chemistry while maintaining a microfluidic platform. PEG microgels were directly functionalized with poly‐L‐lysine and RGD peptides, which evidently enhanced cell adhesion and increased adherent cell populations [86]. Lithographic approaches offer unmatched precision in regulating microgel morphology without oils or surfactants, making them highly attractive for cell encapsulation. However, throughput remains limited by mask or mold dimensions and light source parameters, although faster curing strategies (via higher light intensity or photoinitiator concentration) can partially address this challenge [87].

In conclusion, microgels are commonly fabricated by crosslinking hydrogel precursor droplets, with methods differing in scalability, monodispersity, and biocompatibility. While emulsification, precipitation polymerization, and fragmentation offer simplicity and throughput, techniques such as microfluidics, EHDS, wet‐spinning, and lithography enable precise control over structure and functionality. Beyond the fabrication technique itself, the chosen crosslinking strategy critically shapes microgel properties, cell compatibility, and overall performance.

Albeit the fabrication strategies provide precise control over microgel size, composition, and architecture, their functional relevance is manifested at the global assembly level, where interparticle interactions and packing density govern rheological properties such as jamming and flow behavior, which are underlying features that directly impact the biofabrication process and are inherently linked to physical characteristics of microgels, including size, shape, stiffness, and surface chemistry.

## 3 Microgels in 3D Bioprinting: Leveraging Intrinsic Properties and Jamming Behavior

Among the library of microparticulate biomaterials explored in 3D bioprinting, microgels have recently gained prominence as bioinks, owing to their unique rheological properties and structural versatility, enabling the fabrication of bioactive constructs that address the complex requirements of tissue repair and regeneration [7, 8]. Unlike conventional hydrogel‐based inks, microgel systems exhibit pronounced shear‐thinning and rapid self‐healing behavior, enabling facile extrusion while maintaining construct integrity [20]. This particularity arises from their ability to form cross‐linked hydrogel particles that aggregate into a cohesive bulk construct during extrusion when reaching the jammed state [1]. In this transition, particles are characterized by yield‐stress behavior that allows shape retention immediately after deposition [20, 23]. This section first introduces the fundamentals of microgel jamming, then discusses how different jamming states govern rheology, printability, as well as the structural stability of microgel‐based bioinks, emphasizing their importance in biomedical applications, where spatial precision and long‐term functionality are essential.

### 3.1 Fundamentals of Microgel Jamming

**FIGURE 3:**
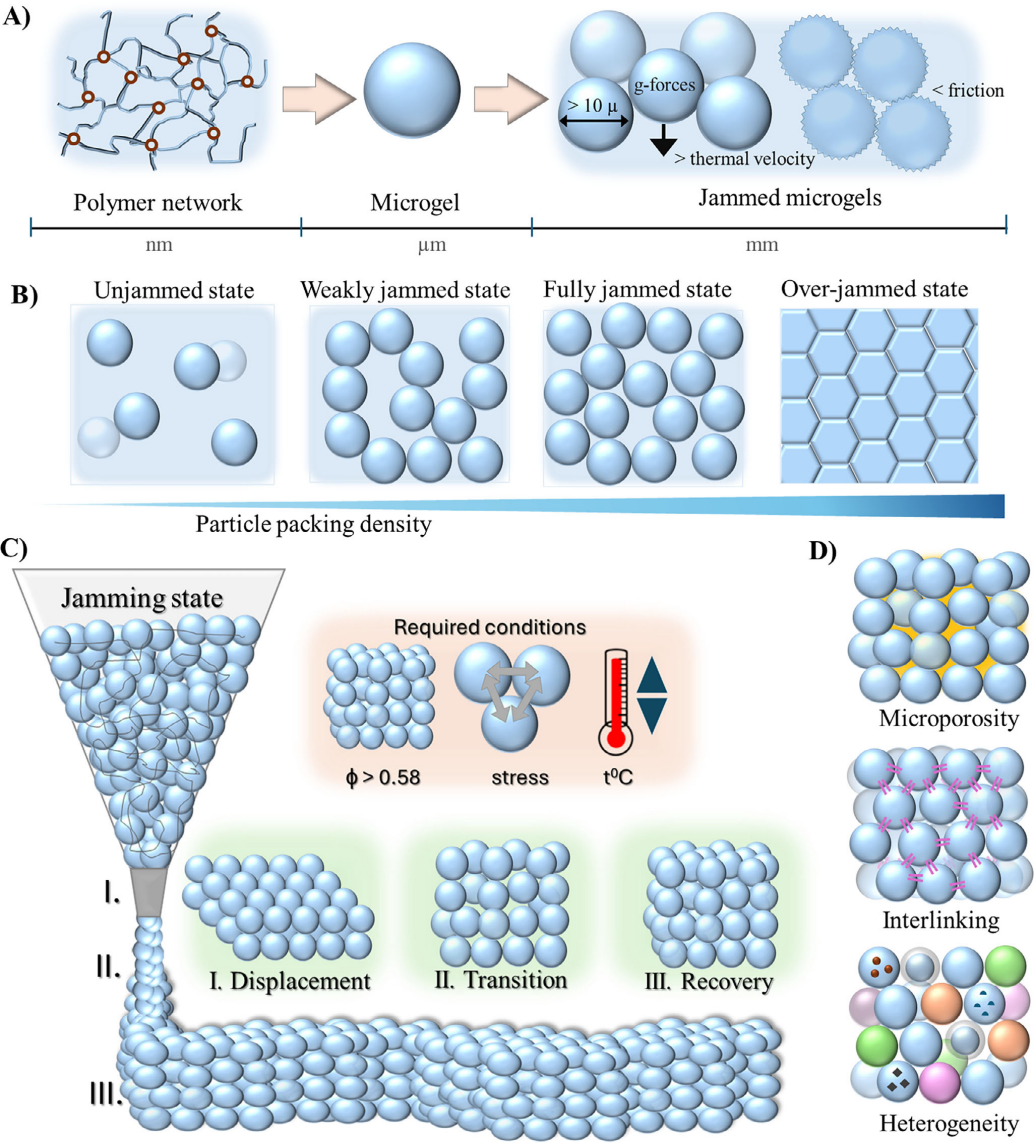
Multiscale structure and functional properties of microgels: (A) Structure and properties of microgels—microgels are characterized by multiscale features: polymer network at nanoscale, individual hydrogel microparticles at microscale, and microparticle assemblies or modular hydrogel at macroscale level. Unlike colloids, microgels are characterized by specific features: size < 10 µm; gravitational forces > thermal forces; (B) Increasing particle packing fraction induces a transition in microgel suspensions from an unjammed liquid‐like state to a weakly jammed state near random loose packing (RLP), followed by a fully jammed granular hydrogel with yield‐stress behavior near random close packing (RCP). Further compression produces an over‐jammed state characterized by very high viscosity and difficult extrusion. (C) Jamming occurs when particles form a percolated network of contact forces and physical constraints that limit individual particle mobility. Gray lines indicate the network of contact forces between adjacent particles. During the extrusion printing process jammed microgels inks undergo three phases: (I) Displacement‐ flowing through the nozzle under shear, (II) Transition‐ adjusting particle interactions as the material yields, and (III) Recovery‐rapidly regaining solid‐like behavior to preserve the printed structure; (D) Inherent and tunable features of microgels: Microporosity: Micron‐sized particles generate pores comparable in size to cells allowing passage through the structure. Interlinking: versatility in interlinking between particles generates a stable structure. Heterogeneity: heterogeneous mixture of particle species, e.g., anisometric particles, porous particles, layered particles, core–shell particles, particles encapsulating cells or small molecules.

Jamming represents an athermal transition between the freely flowing and rigid states in amorphous systems such as granular matter, colloidal suspensions, complex fluids, and even cellular assemblies, which can be induced by modulating different thermodynamic and mechanical variables [88, 89]. In the jammed state, microgels exhibit multiscale structural and functional features that extend from the molecular to the macroscopic level, owing to their complex interactions and condition‐dependent organization [21, 32, 90]. These characteristics are highly dynamic and are governed by particle concentration, external stimuli, and the intrinsic physicochemical properties of the microgels [91, 92] (Figure 3A). Thus, jamming transition of microgels occurs when the particle packing fraction (or particle to volume ratio, ɸ) reaches a critical threshold and under appropriate conditions of stress and temperature [23, 90].

Compared to colloidal‐scaled systems, interparticle interactions of these multifarious soft materials are predominantly governed by strong elastic, gravitational, and frictional forces rather than thermal and van der Waals interactions [90, 92]. Hence, microgels are characterized by a packing‐dependent jamming behavior, transitioning from freely flowing suspensions (unjammed state) to fully jammed granular solids (Figure 3B) [20, 21].

Typically, below the critical packing threshold, microgels are in an unjammed state and behave as freely flowing, independently moving particles in a suspension. As the packing fraction (ϕ) approaches random loose packing (ϕ ≈ 0.58), a fragile contact network appears, and the system reaches the weakly jammed state [20, 93]. In this state, the particles are minimally jammed, the system is characterized by low yield stress, and easy rearrangement of particles, at the same time preserving the global mechanical stability. Increasing the packing fraction toward random close packing (ϕ ≈ 0.64) strengthens interparticle interactions, and the system transitions into a fully jammed state characterized by tightly packed particles that collectively generate a percolated contact network and cause the system to exhibit solid‐like, yield‐stress behavior akin to that of bulk hydrogels [88, 89, 94]. Beyond random close packing, an over‐jammed state may occur (ϕ > 0.64), in which excessive particle interactions along with their deformation and interpenetration increase extrusion pressure and may compromise printability [21, 95].

In fact, soft microgel systems are inherently more complex, primarily consisting of anisometric and non‐spherical particles with high deformability and interparticle friction, which collectively may allow the jamming transition at volume fractions greater than 0.64 [7, 20, 96]. Regardless of their particulate nature, microgel compression during jamming can be controlled using external forces such as centrifugal or mechanical compression [23]. To better understand their behavior, supplementary experiments for examining the packing of anisometric particles with diverse shapes and deformability are performed [69, 90, 97].

### 3.2 Rheological Features and Printability

The rheological properties of microgels, particularly their jamming behavior and shear‐thinning features, play a central role in bio‐fabrication suitability. Shear‐thinning describes the ability of a material to transition between low‐viscosity flow under applied shear stress and high‐viscosity stability afterward, which directly influences printability and shape fidelity [1, 32, 98]. During printing, shear applied in the nozzle causes a transient disruption of interparticle contacts, allowing the material to flow. Once the stress is removed, these interactions are rapidly recovered, re‐establishing yield‐stress behavior (Figure 3C) [20, 21]. This reversible transition originates from the frictional contacts and elastic particle deformation, along with short‐range non‐covalent interactions (including electrostatic interactions) among neighboring particles, which become increasingly significant as particle compression increases [1, 23]. Furthermore, printing performances and shape fidelity are also driven by the physicochemical characteristics of jammed microgel inks: as the size of microgel increases, the printing resolution decreases sharply, and oppositely, smaller diameters retain a better resolution, while a uniform distribution of microgel population can assure the extrusion of smooth continuous filament, unlike an anisometric population, which can lead to irregular deposition [20, 97]. While conventional hydrogels can usually be injected as homogeneous precursor solutions with liquid‐like rheological properties and induce gelation through in situ crosslinking strategies or shear‐tinning mechanisms [7, 23], densely packed microgels can be directly injected owing to their granular nature and small dimensions. Together, the combination of jamming‐induced cohesion, interparticle interactions, and shear‐thinning behavior allows microgels to be easily extruded, forming stable, high‐resolution, cell‐laden constructs.

### 3.3 Structural Stability

Although jamming primarily supports fabrication by conferring both transient structural integrity and a protective environment for encapsulated cells through reducing mechanical damage during the printing process, long‐term mechanical stability and durability of the printed construct are governed by an additional post‐printing step, such as interparticle annealing, naturally achieved by secondary crosslinking (e.g., covalent bonding, photopolymerization, ionic crosslinking, or enzymatic bonding) [20, 21, 99, 100]. Mechanical properties and stability can be tuned via chemical or dynamic interparticle crosslinking [97], generation of a secondary network through interstitial matrices [101], or incorporation of nanoparticles that modify interfacial interactions between microgels [20].

Further, the particulate two‐scale matrix structure of microgels allows them to swell and compress more than bulk hydrogel: first, the nanoscale network within each microparticle, formed by intraparticle polymer crosslinking, governs local swelling, elasticity, and responsiveness; second, the microporous matrix of the jammed state, shaped by interparticle interactions and interlinking among microgels determines bulk compressibility, network connectivity, and mechanical stability [97, 102]. Additionally, the densely packed microgels are also characterized by self‐healing behavior, when their dynamic, reversible interparticle interactions are disrupted [20, 102].

Besides structural properties, the specific multiscale functional features, e.g., heterogeneity, void space/porosity of jammed microgels, should also be considered when designing microgel‐based 3D constructs with proper characteristics for tissue engineering applications (Figure 3D). Since the native tissue is a complex microenvironment featuring numerous structural and biochemical elements that support the interchange of cells and signaling molecules, the heterogeneity is another fundamental feature of microgel inks [103]. The aggregate nature of jammed microgels provides an ideal platform for merging heterogeneity into biomaterials. Integrating intraparticle and/or interparticle heterogeneity can endow the microgel inks with additional complexity that can exercise multifactorial effects at the particle or system level, allowing the building of more biomimetic constructs that better reproduce the native tissue environment [104]. Intraparticle heterogeneity involves the combination of individual particles with adapted surface chemistries, while interparticle heterogeneity implies the combination of various populations of microparticles divergent in a multitude of ways (e.g., formulation, matrix composition, cargo) [1, 23]. Moreover, heterogeneous microgel‐based materials can be formulated by simply mixing multiple species of microgel with various features until particles are uniformly distributed in space; this allows the generation of composite inks that are mechanically anisotropic and functionally heterogeneous [26]. It should be mentioned that increased design complexity brings complex functional outcomes, making it essential to gain a deep understanding of the multifaceted bio‐functionalities that might arise from particle mixing.

The next key functional characteristic of jammed microgels is their ability to form 3D matter with high porosity. The interstitial voids between densely packed microgel particles are directly related to the overall porosity, while the size of these pores is proportional to the size of the assembled microgels (Figure 3D) [105]. Thus, densely packed microgel particles generate 3D matters with micro‐sized pores, which are primordial for their bio‐integration and biological performances. Considering the micron size of most cells, these pores facilitate mass transport and promote tissue integration compared to non‐porous hydrogels [20]. The average porosity of microgel‐based systems is impacted by the geometry, mean diameter, stiffness, and degree of compression of microparticles, while reduced porosity is generally correlated with the decrease in permeability and mass‐transport rates [106].

These rheological and architectural features define the physicochemical environment created by jammed microgel constructs, emphasizing their importance in biomedical fields where architectural precision and long‐term functionality are primordial in the biofabrication process. Particularly, jamming behavior represents the mainstay factor that dictates the shape fidelity and structural integrity, and overall printability performances in extrusion‐based 3D printing. At the same time, this performance directly impacts the cellular behavior, development, and organization as well as tissue formation, which will be discussed in a separate section.

## 4 Crafting Microgel‐Based 3D Constructs: Advanced Strategies for Biofabrication

Successful 3D bioprinting relies critically on the strategic selection of bioinks with appropriate biological, mechanical, and rheological properties to ensure cell viability, tissue maturation, and structural stability. The choice of biomaterial ink directly influences print fidelity, biocompatibility, and long‐term functionality of the construct [107]. Although conventional bulk hydrogels are widely used in bioprinting owing to their ability to recapitulate native tissue architecture, they exhibit limited print resolution and nutrient transport because of their continuous macroscopic networks and nanoscale pores. In contrast, microgel‐based inks combine the tunable mechanical and rheological properties required for accurate deposition with the biological functionality necessary for cell viability and tissue maturation.

Microgels are typically concentrated through centrifugation, filtration, or sedimentation to reach packing fractions sufficient to induce the jammed transition, enabling shear‐thinning and yield‐stress behavior required for extrusion printing. Depending on the formulation strategy, microgels can be used as modular bioinks or dispersed within a percolating continuous hydrogel matrix to form biphasic systems that improve structural cohesion and biological performance. Jammed microgel inks were reported for extrusion 3D printing from both natural (hyaluronic acid, agarose) and synthetic (PEGDA, polyacrylamide) polymers. These systems are usually characterized by suitable rheological properties with yield stress behavior that enables complex 3D designs with improved shape accuracy upon extrusion [23, 25, 108]. Alternatively, microgels can be combined with an interstitial matrix component to generate biphasic bioinks. For example, PEG microgels dispersed within GelMA hydrogels form a biphasic colloidal network with improved rheology, printing fidelity, and structural stability [109]. Cells may also be encapsulated either within individual microgels, distributed in the interparticle space, or embedded in a secondary matrix, allowing flexible control over cell organization and microenvironmental parameters.

**FIGURE 4:**
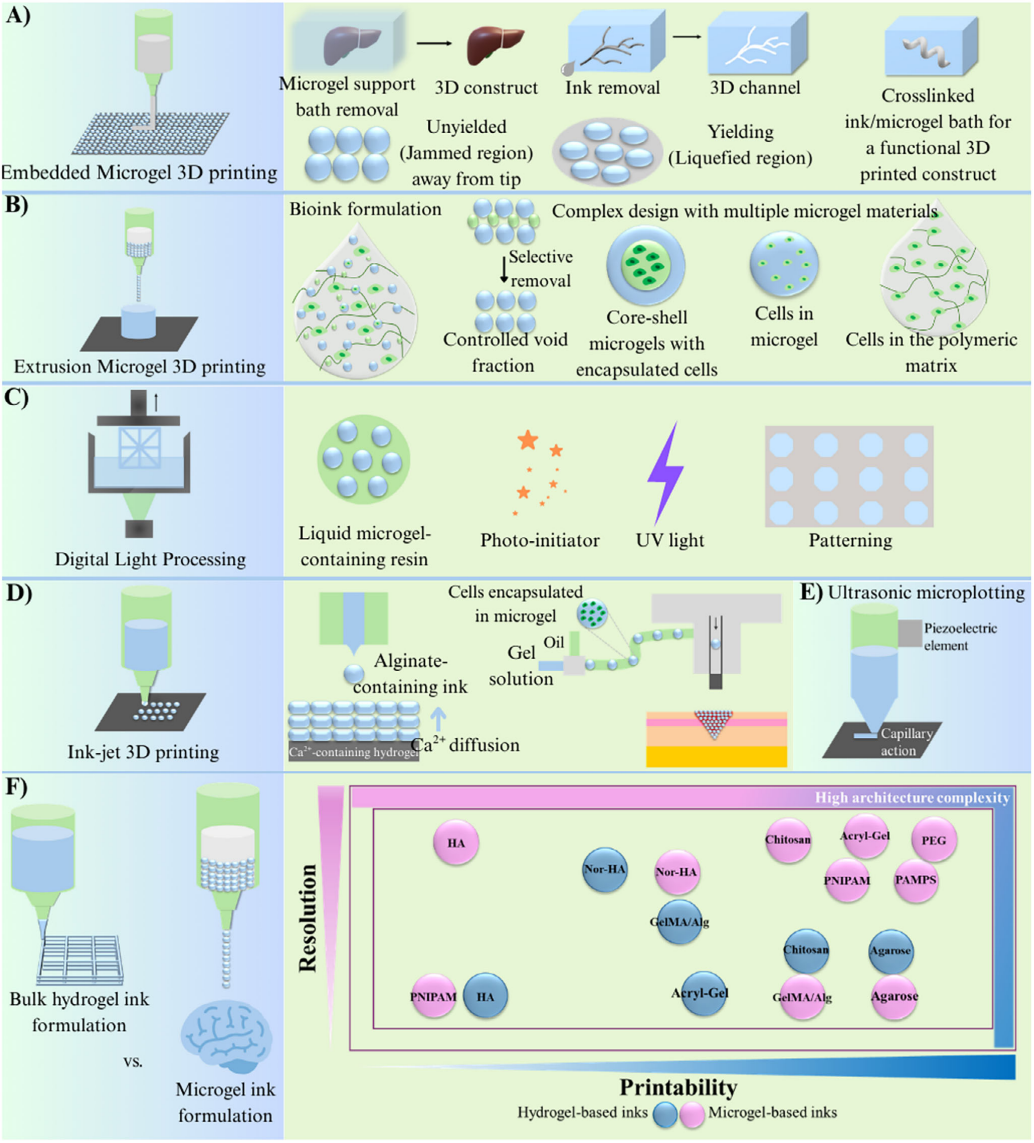
Microgel‐based 3D printing methods for fabricating functional constructs: (A) Embedded 3D printing: printing into supportive microgel baths enables fabrication of intricate, self‐supporting, and perfusable 3D structures within supportive microgel baths, where localized yielding from needle‐induced shear governs print fidelity; (B) Extrusion 3D printing: deposits microgel‐containing ink layer by layer per CAD design; inks may be jammed microgels, polymer‐embedded microgels (with selective removal), cell‐laden microgels, or core–shell structures; (C) Digital light processing: resin with photopolymerizable groups and microgels enables fabrication of complex structures. (D) Ink‐jet 3D printing: tiny droplets of material (ink) are ejected through a nozzle and deposited onto a substrate in a precise pattern; (E) Ultrasonic microplotting uses a piezoelectric element to eject ink beads onto a substrate.; (F) Comparative analysis of extruded‐based 3D printing performance of microgel ink vs. bulk hydrogels (Acryl‐Gel = Acrylate derivatives of gelatin; Alg = Alginate; HA = hyaluronic acid; Nor‐HA = Norborene‐Hyaluronic acid).

Following printing, additional stabilization strategies are usually applied to reinforce the printed construct and ensure long‐term structural integrity, such as interparticle crosslinking or secondary network formation within the interstitial matrix. These versatile stabilization strategies rely on a variety of physical and chemical interactions between neighboring microgel particles, and might include enzymatic reactions, photopolymerization, click chemistry, amine coupling, or non‐covalent interactions such as hydrogen bonding and electrostatic forces [34]. Moreover, these interactions can be tailored to control bioink cohesion and to facilitate the incorporation of bioactive components. Besides stabilizing microgel assemblies, these tunable interactions also strengthen their processability into complex architectures through advanced biofabrication techniques. In this context, approaches such as digital light processing (DLP) and ultrasonic microplotting enable the generation of sophisticated architectures with uniform or gradient microgel distributions [110, 111]. This chapter discusses the main techniques employed for fabricating and printing microgel‐based constructs, highlighting their underlying principles, advantages, and applications in creating complex, biomimetic tissue architectures (Figure 4 and Table 3).

**TABLE 3:** Overview of microgel‐based 3D printing: Properties, applications, and biological performance.

Embedded 3D printing				
Microgel materials	Microgel features	Specific properties	Application	Achievements
Carbopol [113, 114, 115]	Spherical, ⌀ = 7 µm, low concentration bath (0.2%), high stability	Stable complex structures, allowing needle retracing; 3D printing of non‐extrudable PDMS; more homogenous interface between the layers;	Vascular tissue 3D constructs; expanding the array of 3D printable materials;	4 cm DNA model fabricated; uncrosslinked structures stable for 6 months; helical print confirmed interlayer adhesion; tubular structures showed higher strength (54.6 kPa) and >80% cell viability
SEP copolymer S_607_‐b‐EP_1561_ [112]	Spherical, ⌀ = 2–4 µm, optimal bridging for self‐assembly	Optimized rheology for 3D printing: low modulus (100 Pa), yield stress (3–4 Pa), rapid recovery (1 s)	3D printed perfusable networks, functional fluid pumps	High‐resolution (30 µm) 3D vascular network with strong mechanics and efficient fluid flow
PEG [116]	Spherical, ⌀ = 6 µm, loosely packed, enhanced permeability	3D printing of ultra‐low viscosity solutions	Human liver tissue model	ADME behavior mimics the human liver
qDMAEMA, MA [117]	Spherical, ⌀ = 4–5 µm, polyelectrolyte–ion interactions	Tunable rheology: raising polymer charge density raises yield stress	3D cell culture	High cell viability (90%–95% after 24 h) in anionic and zwitterionic microgel baths
Agar [119]	Spherical, ⌀ = 30–130 µm, highly jammed dispersion	Quick self‐healing; microgel bath does not degrade after UV exposure;	3D printing of UV‐curable inks	Stretchable spring 3D‐printed in agar microgel bath; bath supports up to five reuse cycles;
K‐Carrageenan [120]	Spherical, ⌀<1 µm, hydrogen and cationic interactions	Microgel bath with low concentration (0.3%);	Customized tissue and organ engineering	High‐resolution 3D‐printed heart (45 µm) and kidney models supporting 92.1% cell viability and proliferation;
Oxidized and methacrylated alginate (OMA) [121, 122]	Spherical, ⌀ = 7 and 400 µm, photocrosslinked bath resolution	Cell‐only bioinks printable with resolution controlled by microgel size; Tissue formation influenced by methacrylation degree	Complex tissue constructs with various cell types; coronary artery	Microgel bath enables long‐term cartilage‐like tissue culture; smaller needle printing produces robust vascular tissues with aligned collagen fibers after 14 days
Gelatine [123]	Variable shapes, ⌀ = 125–260 µm, enzymatic crosslinked bath	Enhanced printability, multi‐ink 3D printing vs. air;	Perfusable channels for engineered living tissues	3D‐printed lung with alveoli and perfusable channels supporting enhanced cell viability
Extrusion 3D printing				
PAMPS [124, 125]	Spherical, ⌀ = 40–120 µm + inter‐particle adhesion, polyacrylamide percolating matrix	Ink viscosity and rheology are tunable via crosslinker concentration	Soft robotics, implants, and Mechanochromic	3D‐printed shape‐morphing structures with tunable mechanics and 10× higher fracture resistance; color‐changing shapes respond to compression
PNIPAM [126]	Spherical, ⌀ = 150–450 based on t^o^, reversible crosslinks	Thermo‐responsive control of resolution, controlled rheological behavior	4D bioprinting or controlled drug release; Soft actuators	3D‐printed meshes and pyramids show temperature‐ and pH‐responsive changes; complex shapes with high fidelity, stability
PEG [127]	⌀ = 200–600 µm, interparticle cohesion via thiol–ene click chemistry	Stiffer microgels promote cell spreading, increased focal adhesions, and reduced swelling vs. bulk	Tissue engineering	40‐layer cylindrical structure with strong layer adhesion; anatomically relevant ear and nose models printed with high fidelity
Gelatin [8, 98, 128, 129]	Spherical, ⌀<100 µm, hydrophobic‐assisted jamming	Self‐supportive ink	Conductive soft materials; Tissue and organ engineering	Jammed microgels support 57‐layer cylinders; overhangs and anatomical models printed with fine details
	Elongated, ⌀ = 18 µm, sacrificial material	Bacteria encapsulation stiffens the microgel ink; Void fraction controlling	biomineralization tissue engineering	High‐resolution 3D‐printed sphinx with microgel‐mineralized ink; discs with increased porosity enhance cell infiltration
GelMA [130, 131, 132]	Fragmented shapes, ⌀ = 200–600 µm, interdigitation	Stronger microgel interactions increase printability; Adjustable interior porosity	Tissue and liver tissue engineering, wound healing	Enhanced cytocompatibility, proliferation, and vascularization; interconnected network, reconfigurable 3D‐printed objects
Hyaluronic acid [133]	⌀ = 40, 100, 500 µm, secondary crosslinking	Microgel size affects resolution and printing precision	Cartilage tissue engineering	3D‐bioprinted discs exhibit greater mechanical stiffening in vivo vs. bulk
Agarose [134]	Spherical, ⌀ = 1.5 µm, frictional forces, electrostatic interaction	Microgel with self‐healing ability;	Tissue engineering	Stable tall prints with higher 120 h cell survival vs. bulk
Chitosan [135, 136]	Fiber‐like, pre‐crosslinked	High shear recovery; microsphere‐based inks maintain rheology with various monomers.	Tissue engineering	Multilayered 3D prints (ear, nose); microgels speed wound healing; human nose print shows high energy dissipation
Alginate/Collagen [137]	Spherical, ⌀ = 100–200 µm	Cells encapsulated in microgels are protected	Bone tissue engineering	Microgel‐containing ink enhances bone mineral density in in vivo bone defect repair
Gelatine/GelMA [138]	Spherical, ⌀ = 175 µm core–shell, interparticle frictions	Microgels can be used to spatially organize cell communities	Biofabrication and bioremediation	Core–shell microgels support the culture of mammalian cellular spheroids
ADA‐GEL [139]	Spherical, ⌀ = 50–200 µm, contact adhesion (tack) and Ca^2+^ stabilization	Microfluidics‐integrated 3D printing with immediate microgel jamming	Advanced biofabrication	Permeable 3D‐printed constructs with microgel‐derived microporosity enabling efficient fluorescent diffusion
Agarose [108]	⌀ = 100‐120 µm stabilized with a percolating network	Ink with multiple ionic microgel types, tunable for complex spatial configurations	Stretchable and wearable devices	3D‐printed ionic junctions with high toughness and strength; ionic touchpad converts mechanical energy to electricity
Poly(Lys_60_‐ran‐Ala_40_)/PEG [140]	Irregular morphology, ⌀ = 30 µm, polymeric percolating networks	Enhanced printability and biocompatibility	Wound healing	3D‐printed meshes with narrow strand diameter (∼200 µm) spacing (∼500 µm)
Hyaluronic acid, PEGDA, Agarose [23]	⌀ = 100 µm, UV‐light mediated interparticle crosslinking	Combining microgel types does not affect ink extrudability	Advanced biofabrication	Encapsulated cells are viable; perfusable channels are printed with many microgel types
Integrated 3D printed strategy (Embedded & Extrusion)				
Carbopol, Pemulen& PVA & fluorescent microspheres [31]	⌀ = 5 µm (ink)for ink, 1 µm (bath), varying charge and crosslinking densities	High microgel packing lowers osmotic pressure, supporting cells	Tissue engineering	A continuous helical pattern was achieved with a feature size as small as 20 µm
Carbopol/Gelatin & GelMA or Alginate/Gelatin [141]	Irregular shape, ⌀ = 215 µm (ink), 7–30 µm (bath)	Synergistic use of multiple microgel types (as inks or embedded bath) for the fabrication of complex structures	Bioprinting of advanced tissue and organ systems	3D‐printed heart model perfusable with dye; 3D models with higher elastic modulus than cast; hepatic cells proliferate faster
Digital Light Processing (DLP)				
HA functionalized with norborene [142]	Spherical, ⌀ = 20–100 µm, fragmented, ⌀ = 10–300 µm, entanglements of the percolating poly(acrylamide) chains	3D‐printable resins with suitable viscosity and minimal sedimentation	Soft matter applications	Microgels boost strength and fracture work; elastic modulus ∼4× higher than bulk
Gelatin [143]	Spherical, ⌀ = 10, 45, and 100 µm	Gelatine microgels enhance GelMA rheology but impede photocrosslinking	Tissue structures for improved perfusion and diffusion	3D‐printed star structures exhibit improved internal porosity; cells in microgels show 4× higher metabolic activity than bulk
GelMA [144]	Spherical, ⌀ = 10–50 µm in a percolating dextran, annealed assembly	Microgel‐containing resins with suitable flowability	3D cell culture models	GelMA microgel size impacts print fidelity; higher cell activity in microgels than in bulk
Inkjet 3D printing				
Gelatin/Matrigel [145]	Spherical, ⌀ = 450–650 µm, fusing in contact with support	Granular gel may be applied directly to the muscle lesion in vivo	Skeletal muscle and hair follicle regeneration	Microgel‐based constructs promote superior tissue regeneration (muscle and skin) vs. bulk hydrogels or cell suspensions
Alginate [146, 147]	Spherical, ⌀ = 40 µm	Microgel size tunable via nozzle; alginate droplets crosslink without spreading for high‐resolution printing	3D biofabrication of tissues, organs, and microvasculature	Microscale 3D prints (200 µm tube); bifurcated vasculature with perfusable capillaries
pNIPAm [148]	prepolymerized colloidal particles	Print accuracy rose by increasing microgel concentrations in the inks.	3D‐printed structures for modulating cells	3D printed features as small as 50 µm

*Abbreviations*:; ADA‐GEL—Alginate/ poly(ethyloxazolines)/poly(methyloxazolines)/ pentenoic acid modified gelatine; HA—hyaluronic acid; MA—methacrylic acid; PAMPS—Poly(2‐acrylamido‐2‐methylpropane sulfonic acid); PNIPAM—Polyacrylamide/poly(N‐isopropylacrylamide);qDMAEMA—Quaternized 2‐(dimethylamino)ethyl methacrylate, carboxybetaine methacrylate; SEBS—polystyrene‐block‐ethylene/butylene‐block‐polystyrene triblock copolymer; SEP Polystyrene‐block‐ethylene/propylene diblock copolymer.

### 4.1 Embedded 3D Printing

Embedded bioprinting uses a jammed microgel support that fluidizes under shear during printing and rapidly resolidifies upon stress removal (Figure 4A and Table 3). This allows high‐resolution, controlled additive manufacturing owing to the microgel's low modulus, yield stress, fast elastic recovery, and localized yielding [112]. Carbopol‐based microgels, among the first support materials employed in embedded 3D printing owing to their yield‐stress and thixotropic properties, were established as a benchmark by Bhattacharjee [113], who demonstrated their ability to fabricate complex structures, such as a 3D DNA model. Hinton et al. [114] later demonstrated 3D printing of otherwise challenging materials like polydimethylsiloxane within Carbopol microgels into a cylindrical structure, while a concentration of 0.8% (w/v) Carbopol at neutral pH was identified as optimal for printing alginate [115]. Beyond Carbopol, synthetic polymer‐based microgels, such as PEG, diblock/triblock copolymers, and polyelectrolyte systems incorporating cationic, zwitterionic, or anionic groups, have been explored as tunable support baths for embedded printing [112, 116, 117]. Hybrid microgels combining natural and synthetic polymers (e.g., gelatine–alginate–PVA) have also been developed for tissue‐specific applications [118]. On the other hand, natural polymer‐based supports, including agar and k‐carrageenan microgels, have demonstrated remarkable self‐healing and load‐bearing capabilities [119, 120, 121, 122, 123]. Firmly compacted agar microgels (> 0.5% w/v) enabled 3D printing of hollow helical and triangular structures [119], while k‐carrageenan sub‐microgels (∼ 642 nm) at concentrations as low as 0.3% (w/v) facilitated the bioprinting of organ‐like constructs [120].

### 4.2 Extrusion 3D‐Printing

Extrusion 3D printing enables patient‐specific fabrication of complex tissues, organs, and medical devices by layer‐by‐layer deposition of biomaterial inks based on CAD models. Print fidelity and precision are controlled through parameters such as printing speed, pressure, nozzle diameter, and ink rheology [19, 107, 149, 150] (Figure 4B and Table 3). It is reported that various physico‐chemical properties of 3D printed constructs are improved by using micrometric particles. For instance, among synthetic systems, polyelectrolyte‐based poly(2‐acrylamido‐2‐methylpropane sulfonic acid) (PAMPS) microgels had 10‐fold higher mechanical resilience vs. their bulk counterparts, and their versatile composition enabled stimuli‐responsive crosslinking for applications in soft robotics and implants [124, 125]. Moreover, Zheng et al. [151] demonstrated the robustness of microgel‐based inks by 3D printing a shoe insole capable of supporting 1 kg, while Charlet et al. [152] showed that PAMPS/polyacrylic acid inks can be fully recycled with ∼100% yield. pNiPAM microgel inks have also demonstrated their feasibility to build stimuli‐responsive designs, showcasing on‐demand fragmentation and temperature‐triggered resolution enhancement [126]. The size of microgels can also impact the final 3D construct, as observed by Guan et al. [127, 153, 154]. Collectively, these synthetic microgels offer tunable rheology, mechanical robustness, and functional responsiveness, supporting complex 3D bioprinting applications.

Natural polymer‐based microgels, particularly gelatin, GelMA, hyaluronic acid, agarose, and chitosan, are widely used for biocompatible 3D bioprinting [128, 129]. For instance, Sheikhi et al. [98] developed a gelatine‐based jammed microgel system using deep eutectic solvent‐based crosslinking (L‐arginine and glycerol), and they achieved the fabrication of tall, unsupported architectures, surpassing conventional high‐aspect‐ratio hydrogels prone to collapse. In another work, Seymour et al. [8, 130] used gelatin microgels as sacrificial components in GelMA‐based granular inks to create interconnected porous structures with enhanced cell infiltration. In addition to functioning as a bulk hydrogel matrix, GelMA can be processed into microgels, as proved by Fang et al. [131], who created a cell‐embedded bioink containing C2C12 cells within a GelMA precursor, promoting high cell viability and enabling complex structures. Dynamically crosslinked microgel bioinks further improve interparticle interactions, printability, and cell infiltration, with L929 cells migrating up to 425 µm into printed structures [132]. Hyaluronic acid microgels achieved high‐resolution (40 µm) ear‐like and lattice structures with superior mechanical strength and uniform glycosaminoglycan and collagen I/II deposition compared to bulk hydrogels [133], while agarose‐based microgel bioinks improved cell survival over 120 h compared to bulk hydrogels, promoting mass transport through their microporous networks [134].

Chitosan is another promising material for microparticulate bioinks [135, 136]. Zhang et al. developed a tunable chitosan microsphere‐based bioink, controlling composition and mechanics via microsphere swelling, and printed an octopus model with bilayer tentacles and a pNiPAM head that coiled upon hydration, while chitosan enhanced cell adhesion. Other microparticulate inks include buckwheat protein, carboxylated cellulose nanofibers [155], and blood‐derived proteins (platelet lysate, albumin) [156].

To overcome the limitations of classical microgel fabrication, typically time‐consuming, low‐yield, and multi‐step, Reineke et al. [139] proposed an automated strategy combining microfluidics for microgel production with 3D bioprinting for scaffold fabrication. Other approaches focus on complex ink formulations combining natural and synthetic polymers; for example, Huo et al. [108] used ionic microgels to print functional ionic devices, while hybrid microparticulate inks improved printability and resolution of otherwise non‐printable formulations [23, 140]. Emerging strategies also combine embedded microgel printing with extrusion‐based methods. Fang et al. [32, 141] demonstrated this by successive printing in reversible ink templates to create cell‐rich biphasic bioinks with perfusable ventricular models.

### 4.3 Digital Light Processing (DLP)

Digital light processing (DLP) is a high‐precision, light‐based 3D printing method that fabricates complex structures layer‐by‐layer using photo‐crosslinkable materials [151] (Figure 4C and Table 3). Crosby et al. [142] demonstrated its potential by embedding hyaluronic acid microgels in an acrylamide matrix to 3D print intricate geometries, achieving up to a 4‐fold increase in stress at failure at 50% v/v microgel loading. In another work, Ouyang et al. [143] designed a gelatine microgel–GelMA composite bioink, producing microporous hydrogels with tunable porosity (20%–70%) and pore sizes as small as ∼10 µm, which were successfully printed into complex, high‐aspect‐ratio structures with enhanced integrity compared to pure GelMA. For bioprinting applications, Wang et al. [144] developed a multicomponent bioresin comprising dextran, methacrylated galactoglucomannan, and GelMA microgels; dextran dissolution during cell culture generated microporosity, promoting cell proliferation and resulting in higher viability than bulk hydrogels.

### 4.4 Bead‐ or Ink‐Jet Printing

Ink‐jet (bead‐jet) printing enables precise deposition of cell‐laden microgels for high‐resolution tissue engineering, allowing fabrication of small, well‐ordered structures (Figure 4D and Table 3). The potency of this method is exemplified by Cao et al. [145], who developed an in situ ink‐jet 3D printing approach using Matrigel microgels loaded with mesenchymal stem cells (MSCs), demonstrating enhanced regeneration of skeletal muscle and skin, with minimal fibrosis, hair follicle formation, and thicker dermal layers. In a related work, Nakamura et al. [146] fabricated viable 3D tubular tissues (∼200 µm diameter) from ∼10 µm cell‐laden alginate microgels within a CaCl_2–_gelatin support medium. Despite high spatial precision, ink‐jet systems remain limited by viscosity handling and droplet uniformity.

Building upon jet‐based printing technologies, ultrasonic microplotting (UMP) has emerged as a sophisticated technique that employs acoustic energy for high‐precision, contactless deposition of microgels and bioinks, allowing the generation of complex, cell‐friendly 3D structures (Figure 4E). Using a piezoelectric actuator connected to a glass capillary writing head, UMP prints colloidal microgel solutions with minimal shear stress. For instance, aiming to overcome the limitations of layer‐by‐layer techniques, Chester et al. [148] explored ultrasonic microplotting to precisely deposit microgel films, fabricating scaffolds with tunable mechanical, adhesive, and bioactive properties.

Most of the additive manufacturing techniques relevant to hydrogel‐based inks can also be used for microparticulate inks, and the choice of 3D printing method depends primarily on the characteristics of the ink (e.g., viscosity, packing, cell content, stability, and self‐healing properties) or on how the printing process is optimized to accommodate specific microparticulate materials. When using low‐viscosity or cell‐only inks in embedded 3D printing, microgel baths facilitate the fabrication of intricate structures by extending the printability window and enabling the deposition of cell‐only filaments. Unlike bulk hydrogel support baths, microparticulate ones rely on the reversible jamming behavior of microgels, allowing multiple needle retracing and enabling the formation of vascularized tissue constructs. In contrast, hydrogel‐based support baths generally rely on polymer crosslinking, and repeated needle movement might disrupt the network and reduce shape fidelity. In microgel baths, physical interactions are often sufficient to stabilize deposited filaments, enabling the formation of perfusable constructs owing to ease of bath removal.

In extrusion 3D printing, higher‐viscosity inks are typically used because they are optimized to flow under shear stress and rapidly recover their structure after withdrawing the nozzle from the printing platform. Since the polymeric layer shields cells from shear stress, this technique is particularly suitable for printing cell‐laden materials. In addition, many biocompatible materials can be adjusted to exhibit excellent printability, making extrusion‐based printing highly cytocompatible. The inclusion of microparticulate components can significantly improve filament formation stability and printing fidelity compared with conventional hydrogel inks, enabling the fabrication of more complex structures.

Figure 4F displays a comparison between natural and synthetic materials used for extrusion 3D printing in both bulk hydrogel and microgel forms in terms of printability, resolution, and architectural complexity. For instance, gelatin and chitosan derivatives, agarose, and PNIPAM microparticulate inks exhibit superior printability and resolution compared with their hydrogel counterparts. Furthermore, several non‐printable hydrogel materials (e.g., PAMPS and PEG) become printable in microgel form. Microgel‐based inks can also simplify the complexity of printing conditions; for example, agarose microgel inks no longer require heated mantles or cooled printing supports. Integrated printing strategies (embedded and extrusion) provide strong filament support and allow the deposition of multiple layers, enabling the formation of structures with high feature resolution and complex geometries. Complex shapes can also be obtained using DLP; however, the biomaterial ink must be photocrosslinkable, which limits the available material collection. For example, varied topologies such as structures with knotted loops, which are challenging to fabricate with traditional molding techniques, can be readily printed with high resolution. For specific applications such as stem cell therapy, ink‐jet 3D printing has also been reported because it can be coupled with microfluidic devices to deposit high cell‐density microgels produced on demand.

To summarize, microgel‐based 3D printing offers a versatile toolbox for biofabrication, with each technique balancing unique strengths and limitations. Embedded printing excels in supporting soft inks and complex architecture, yet requires a supporting matrix that may complicate post‐processing, while extrusion enables high cell densities and large constructs but often faces limitations in resolution and filament fidelity. DLP leverages photopolymerization to fabricate rapid, high‐resolution structures, although it is restricted to photo‐crosslinkable materials and may induce phototoxicity in sensitive cells. At the same time, bead‐ or ink‐jet printing allows precise microgel placement; however, it is constrained by ink viscosity and droplet uniformity. Ultrasonic microplotting further enables non‐contact, high‐viability deposition with exceptional spatial control, though specialized equipment may limit scalability. Together, these methods enable the fabrication of complex, functional constructs for tissue engineering, regenerative medicine, and drug delivery, while the selection of the 3D printing approach is mainly dictated by the resolution requirements, bioink properties, and design complexity. However, outside printability and architectural control, these parameters play a pivotal role in defining the cellular microenvironment. Specifically, the design and assembly of microgels into printable bioinks directly impact cell distribution, cell–material interactions, and nutrient diffusion, and ultimately govern cellular responses and tissue development, highlighting the central link between biofabrication and biological performance, which will be addressed in detail in the following section.

## 5 From Ink to Life: Biological Integration of Microgel‐Based 3D Constructs

The relationship between biomaterials and biological systems is a critical axis upon which the success of tissue engineering and regenerative medicine pivots [157]. While advancements in fabrication techniques and material design have yielded increasingly sophisticated constructs, it is ultimately the biological performance, the material's capacity to support, direct, and integrate with cellular and tissue‐level processes, that determines its translational viability [158]. A comprehensive understanding of the cellular responses to biomaterials, including viability, adhesion, proliferation, differentiation, and immunological interactions, is indispensable for guiding the rational design of functional and clinically applicable scaffolds [157, 158, 159].

Although microgel‐based 3D printed constructs are considered among the most compelling advancements in biofabrication owing to their amenability in constructing architecturally complex cell‐responsive, biomimetic structures [25, 34], the biological behavior remains an area of intense investigation. Their multiscale architecture permits not only the encapsulation and spatial organization of cells, but also the localized presentation of bioactive moieties, such as peptides, proteins, or ECM‐derived ligands, which collectively modulate cell fate decisions [160]. Importantly, the biological interface of these materials extends beyond the cellular level, encompassing interactions with the innate and adaptive immune systems, which govern the tissue integration, remodeling, or rejection of the implanted construct [160, 161].

Emerging evidence has demonstrated that microgel‐based systems can foster diverse biologically relevant outcomes: angiogenic sprouting of endothelial cells [162], osteogenic differentiation of mesenchymal stem cells [163], tumor‐mimetic behavior in cancer models [22], and macrophage‐mediated immunomodulation [164]. In vivo studies further corroborate these findings, revealing differential patterns of vascularization, fibrosis, and immune cell infiltration, depending on the physicochemical features of the microgels and the formulated bioink [99, 165].

1 **Box 2 | A microgel‐based platform for controlled mechanostimulation of stem cells**

Delivering precise mechanical cues in 3D environments remains challenging. Dr N. İyisan and colleagues at the Technical University of Munich developed 3D‐PRESS (3D‐Printed Pressure Chamber for Encapsulated Single‐Cell Stimulation), a 3D‐printed pressure chamber enabling controlled compressive or hydrostatic stimulation of single stem cells encapsulated in microgels [164, 165].

***Device design and methodology***: The system combines RGD‐functionalized alginate microgels with a transparent, pneumatically actuated chamber sustaining pressures up to 400 kPa and compatible with live‐cell imaging. Uniform, biocompatible microgels generated via microfluidic droplets maintain MSCs viability under tunable loading and allow high‐throughput mechanostimulation [164, 165].

***Key findings and biomedical importance***: Controlled mechanical stimulation within 3D‐PRESS activates mechanosensitive pathways, including intracellular calcium signaling and YAP nuclear translocation [164]. Extending this approach, cyclic hydrostatic pressure applied to single MSCs within viscoelastic microgels was sufficient to induce osteogenic differentiation in the absence of biochemical cues—evidenced by RUNX2 and ALP expression, collagen I production, and matrix mineralization over 21 days [166]

By combining precise force control with a biologically relevant microenvironment, the platform enables dissection of mechanotransduction at single‐cell resolution and paves the way for designing mechanically “preconditioned” cells for regenerative and tissue‐engineering applications.

In light of the growing scientific attention dedicated to the biological performance of microgel‐based 3D printed constructs, this chapter aims to elucidate the multifaceted biological responses to these architecturally and chemically tunable biomaterials, thus establishing a conceptual framework to inform next‐generation design strategies that advance therapeutic efficacy, foster immune compatibility, and enhance translational readiness [157, 158, 165]. Many studies have investigated these aspects from complementary perspectives, including cellular viability and functional performance after bioprinting, tissue integration and ECM remodeling both in vitro and in vivo, and the spectrum of immunological responses triggered upon implantation.

**FIGURE 5:**
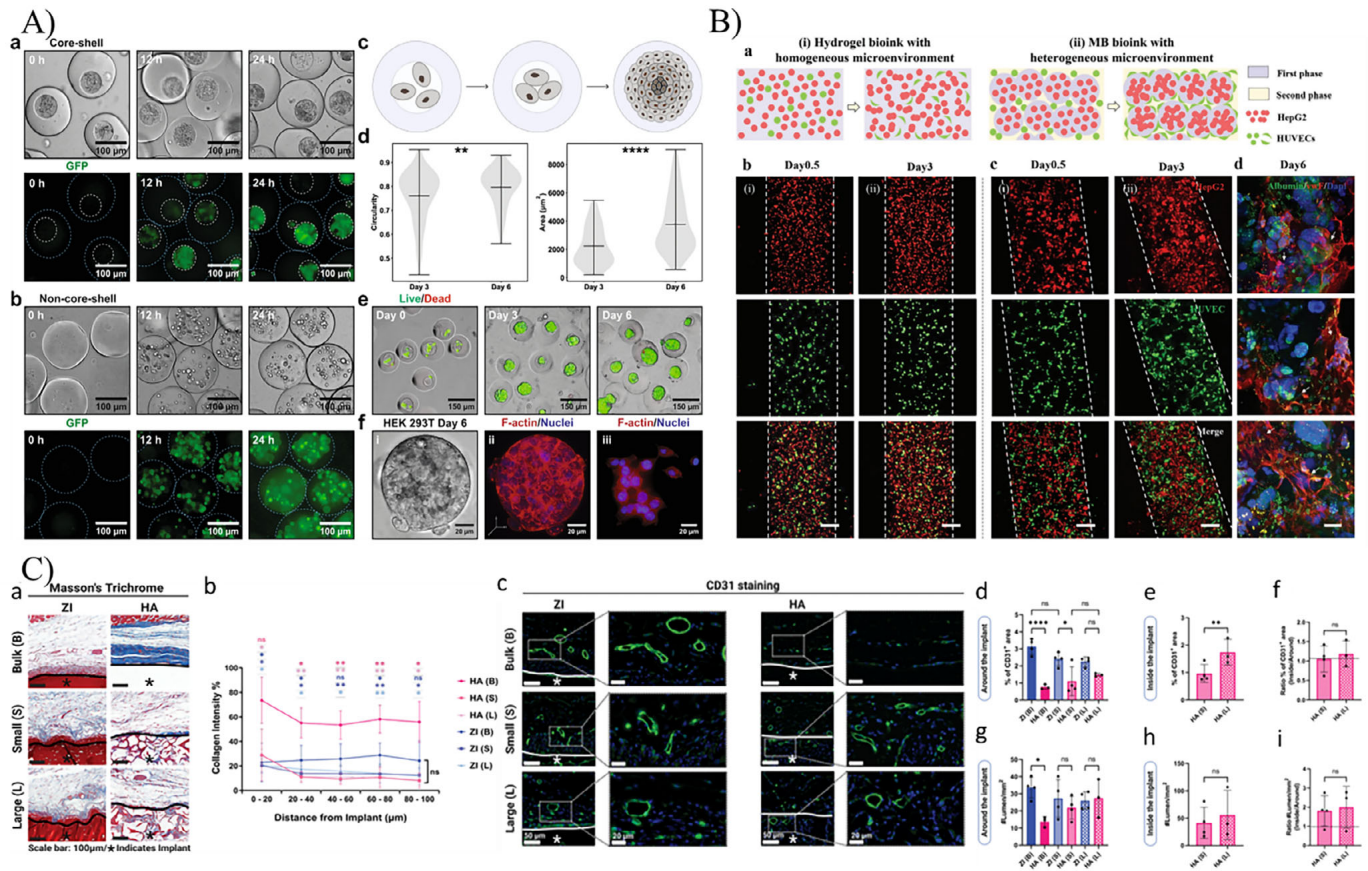
Multifaceted biological outcomes of microgel‐based 3D printed constructs. (A) Core–shell microgels for cell culture—(a,b) GFP *E. coli* proliferation in core–shell vs. non‐core–shell microgels, (c) Schematic of multicellular spheroids formation, (d) HEK 293T spheroids growth (day 3–6) quantified by circularity and area, (e) Live/Dead staining of cell‐laden microgels (days 0, 3, 6), (f) Cytoskeletal structure of HEK 293 T spheroids. (i) Brightfield (ii) confocal (iii) monolayer control. Reproduced (Adapted) from [138] under the terms of Creative Commons Attributions 4.0 International License (CC BY4.0); (B) 3D printed vascularized hepatic tissue constructs with tunable heterogeneous microenvironment—(a) Schematic of spatial arrangement of HepG2 and HUVECs in hydrogel and MB bioinks, (b,c) fluorescence images of HepG2 (red) and HUVECs (green) on days 1 and 3 using (b) hydrogel bioink and (c) MB bioink, (d) Immunostaining of albumin (HepG2) and vWF (HUVECs) on day 6 showing vessel formation (white arrows). Reproduced (Adapted) with permission from [131]. Copyright 2021, JohnWiley &Sons Inc. (C) In vivo performances of zwitterionic granular hydrogels—(a) Masson's trichrome staining showing fibrotic collagen (blue) around implants at 10 weeks, (b) Quantified collagen density (≤100 µm from interface), (c) CD31 immunostaining of vascularization (interface line, * indicates implant), (d,e) CD31+ cell quantification around and inside implants, (f) Ratio of CD31+ cells inside vs. around hydrogels, (g,h) Lumen density adjacent to and within implants, (i) lumen ratio inside vs. around HA granular hydrogels. Reproduced (Adapted) from [168]. under the terms of the Creative Commons Attribution 4.0 International License (CC BY4.0).

Cellular viability and functional performance following bioprinting represent primary determinants of the success of microgel‐based 3D constructs, as the printing process itself can impose mechanical, thermal, and chemical stresses capable of compromising cell integrity. Recent investigations have demonstrated that the intrinsic properties of microgels, e.g., particle size, crosslinking density, viscoelastic behavior, and surface chemistry, play pivotal roles in safeguarding cell viability during extrusion while supporting post‐printing proliferation, migration, and differentiation. For instance, Highley et al. [23] developed several types of jammed microgel inks and evaluated cell viability following both encapsulation and printing. Live/Dead assays showed sustained viability of 3T3 fibroblasts (∼70%), indicating that extrusion did not enforce significant mechanical damage to cells. Furthermore, this strategy allows the precise modulation of the cellular microenvironment through tailored microgel design without relying on additives that could disrupt cell behavior. Similarly, Zhang and co‐workers [166] fabricated diverse 3D constructs from methacrylated chitosan and PVA hydrogel microparticles with outstanding self‐support and sophisticated structures that effectively sustained the growth of bone‐marrow‐derived mesenchymal stem cells (BMSCs) and spheroids. In another study, Pal et al. [167] showed that cell‐laden microgel bioinks composed of gelatine methacryloyl (GelMA), alginate, or their combination maintained > 90% viability and proliferation of MDA‐MB‐231 cells and human dermal fibroblasts over seven days in both encapsulation and scaffold fabrication, with GelMA microgels exhibiting the most favorable performance. Recently, Ou et al. [138] developed microfluidic‐based bioprinting strategies employing protein‐based core–shell microgels, in which a viscous, cell‐laden core was encapsulated by a stabilizing hydrogel shell. This architecture protected microbial and mammalian cells from shear‐induced damage during extrusion, enhanced nutrient and oxygen transport, and sustained high cell viability and long‐term metabolic activity. The construct supported phenotype‐specific ECM production in mammalian spheroids and enabled cooperative interactions within microbial cultures. Furthermore, spatially segregating distinct cell types within individual microgels preserved cellular compartmentalization while allowing paracrine signaling, underscoring the capacity of core–shell microgels to provide mechanically protective yet biologically permissive environments for complex living systems. These findings highlight the ability of microgels to provide mechanically protective yet biologically permissive microenvironments for complex living constructs (Figure 5A).

Beyond supporting cell viability, microgel‐based 3D constructs must integrate with host tissues and direct ECM remodeling to achieve functional repair. Tissue integration involves coordinated cell adhesion, migration, and communication, while ECM remodeling comprises dynamic synthesis, organization, and degradation of matrix components in response to biochemical and biomechanical signals, processes which in microgel systems are governed by their microscale architecture, porosity, and biochemical functionalization [169]. In vitro models reveal early matrix deposition and cell–material interactions, while in vivo studies capture the complexity of vascularization, immune modulation, and long‐term remodeling, primordial features in designing scaffolds that transition into fully integrated and functional tissue [170].

It was demonstrated that a biomimetic alginate‐based 3D microgel model of multiple myeloma can promote tumor cell survival and drug resistance by mimicking the bone marrow niche, providing a robust platform for studying cancer–microenvironment interactions and testing therapies in a physiologically relevant setting [171]. Aiming to tackle the common trade‐off between printability and biological functionality, Fang et al. [131] introduced a cell‐laden microgel‐based biphasic (MB) bioink, combining densely packed microgels with a hydrogel precursor network. The formulated microgel‐based biphasic bioink was capable of creating a heterogeneous but supportive microenvironment that can spatially organize different cell types within a single printed construct. Using hepatocytes and endothelial cells as models, the system promoted cell viability, spatial organization, and functional maturation, with endothelial cells forming vascular‐like networks that enhanced hepatocyte function. The biphasic architecture also facilitated nutrient and waste exchange, mimicking key aspects of native tissue microenvironments and enabling complex tissue models with improved physiological relevance. The authors reported that the printed hepatic tissue sustained high cell densities (up to 50 million cells/mL), with spatial patterning in the MB bioink accelerating cellular reorganization, vascularization, and functional maturation, resulting in enhanced hepatic activity (Figure 5B). Min et al. [118] developed a microgel‐assisted 3D printing strategy using an alginate microgel bath to fabricate high‐resolution decellularized extracellular matrix (dECM) nerve grafts reinforced with a polycaprolactone (PCL) conduit. In a rat sciatic nerve defect model, the printed grafts exhibited robust axonal regeneration, reinnervation of target muscles, and functional recovery comparable to autologous nerve grafts, the current clinical gold standard. Then histological analyses revealed dense axonal growth, organized myelination, vascularization, and ECM remodeling, indicating successful host–graft integration and demonstrating the regenerative potential of microgel‐assisted 3D printed nerve grafts. Further, Kajtez et al. [172]developed an embedded 3D printing approach using human neural stem cells in self‐healing annealable particle–extracellular matrix (SHAPE) composites to promote tissue integration and ECM remodeling in neural tissue engineering. Constructs composed of alginate microgels within a collagen‐based ECM enabled precise neural patterning, supported stem cell differentiation into neurons, and promoted axonal outgrowth and interregional connectivity over several weeks. Lee et al. developed shear‐thinning, self‐healing granular microgel hydrogels that support high cell viability during 3D printing [173]. Biologically, these hydrogels maintain high cell viability post‐printing by protecting cells from mechanical stress and providing a porous, supportive environment conducive to cell proliferation and unction. Besides, their modular design enables controlled therapeutic delivery, enhancing regenerative potential, and highlighting the potency as versatile, cell‐friendly bioinks for tissue engineering and drug delivery.

The immunological response to implanted microgel‐based 3D materials significantly impacts their integration, longevity, and therapeutic performances. Upon implantation, these constructs interact dynamically with the host immune system, triggering cascades of cellular and molecular events that can either promote constructive remodeling or provoke adverse inflammation and rejection. Understanding this complex immune landscape is utmost for designing microgel‐based 3D matters that support tissue regeneration and minimize rejection. For instance, Asadikorayem et al. [168] investigated immune responses to microporous microgels composed of zwitterionic carboxybetaine acrylamide and hyaluronic acid, revealing material‐ and porosity‐dependent effects on in vitro immunostimulation and in vivo fibrosis. The authors noted two distinct immune responses toward the two types of microgels. Zwitterionic (ZI) microgel‐based matters were encapsulated by an inflammatory cell layer, resisting cell infiltration, and could be used as carriers or fillers where host invasion is undesirable. In contrast, hyaluronic acid (HA) microgel‐based scaffolds allowed cell and tissue infiltration, enhancing cell and vasculature ingrowth, highlighting their potential for tissue repair. In a 10‐week subcutaneous mouse model, both ZI and HA scaffolds reduced fibrotic capsule formation and enhanced tissue ingrowth and vascularization compared to bulk HA hydrogels, key factors for successful in vivo integration and long‐term performance (Figure 5C). Another study by Griffin et al. [174] investigated the immune responses to microporous annealed particle (MAP) scaffolds formed by microgels. According to the results, these constructs can direct a regenerative type 2 immune response, reduce fibrosis, and enhance tissue repair, highlighting the potential of MAP scaffolds as effective immunomodulatory platforms.

In summary, current research on microgel‐based 3D constructs is advancing outside proof‐of‐concept stages into disease‐relevant animal models, demonstrating key features such as functional outcomes, vascularization, immune compatibility, and tissue‐specific ECM remodeling, which are critical milestones for eventual translation to clinical trials.

## 6 Engineering Living Systems: Functional Applications of Microgel‐Based 3D Printed Constructs

Native tissues are often characterized by intricate, repeating microarchitectures that are critical for their function. To reconstruct these complex spatial organizations, tissue engineering and regenerative medicine strategies must go beyond conventional scaffold fabrication, which typically offers limited control over cell positioning, vascularization, and hierarchical structural features [32]. To address these limitations, advanced additive manufacturing approaches have emerged that leverage microgel‐based biomaterial inks. These inks combine excellent flow through fine nozzles with tunable porosity, stiffness, and the ability to incorporate a variety of bioactive cues [24, 34]. Unlike traditional bulk hydrogels, which form millimeter‐scale constructs with disordered and heterogeneous micropores, microgel‐based systems allow precise engineering of microarchitectures at high resolution with controlled porosity and stiffness. This encourages not only sustained cell survival and organized tissue formation but also the recreation of tissue‐specific microenvironments, ultimately driving more effective and functional regeneration [25, 34].

This chapter highlights the versatility and wide‐ranging applications of microgel‐based 3D constructs, emphasizing their contribution in advancing various biomedical fields, such as tissue engineering, regenerative medicine, and drug delivery, as well as advanced biofabrication and soft robotics. Additionally, Table 4 summarizes representative advanced microgel‐based 3D constructs along with their biological performance evaluated through both in vitro and in vivo studies.

**TABLE 4:** Transformative applications of microgel‐based 3D printed constructs.

Application	Microgel architecture	Polymeric matrix	Loaded agent	Microgel fabrication technique	In vitro performance	In vivo performance	Ref.
	Bone and cartilage tissue engineering						
	Simple	OMA	—	Mechanical blending	MSC aggregates form cartilage‐like tissue with hMSC maturation and ECM deposition	—	[121]
	Bioactive	Tyramine‐modified HA	Human auricular chondrocytes	Mechanical sizing of bulk gels through meshes	High cytocompatibility; Cell viability >90% for 21 days in vitro; Development of mature fibrocartilage tissues; Intense opacification and mechanical strengthening of the cell‐filled 3D printed scaffold	40 µm microgels showed excellent in vivo integration and stability; Cartilage matured by 63 days; Explanted scaffold exhibited 237 kPa modulus	[133]
	Core–shell	Type I collagen/alginate SilMA/GelMA	BMSCs	Multichannel microfluidics	Enhanced cell viability and proliferation in the microgel‐containing group compared to microgel‐free controls after 7 days, with higher SilMA content reducing the in vitro degradation rate	Good biocompatibility in vivo and better bone repair efficiency after 2 weeks’ implantation in the skull defects of rats	[137]
	Cardiovascular tissue engineering						
	Simple	OMA	—	Mechanical blending/coaxial microdroplet	3D printed rat aorta smooth muscle cells align radially and elongate, creating self‐supported vascular‐like tissues after 2 weeks	—	[122]
		Carbopol	—	Commercial product	A bifurcated cellular tube was obtained with >80% fibroblast proliferation		[115]
	Bioactive	Alginate/Gelatine/GelMA + Carbopol/Gelatin	hiPSCs/HepG2/ NRVMs	Flow focusing microfluidic process	After 14 days, functional cardiac organoids formed, and luminal perfusion markedly enhanced cell survival	—	[141]
	Complex/solid organ tissue engineering						
	Simple	PEG	—	Inverse emulsion polymerization	3D printed tissues show higher urea and albumin production than spheroids	—	[116]
		Kappa–Carrageenan	—	Mechanical grinding	Remarkable viability >92.1% and robust growth of bone marrow MSCs organs	—	[120]
	Other Tissue Engineering Applications						
	Simple	HA/PEGDA/agarose	—	Microfluidics	Viability ∼70% of NIH 3T3 fibroblasts after encapsulation within the microgels and printing jammed microgel inks	—	[23]
		GelMA/gelatin	—	Complex coacervation	High HUVEC viability (>95%) at days 1 and 7, with evident migration and infiltration into 3D printed constructs	—	[8]
	Bioactive	PL/BSA	hASCs	Mechanical fragmentation	Initial cell death within 24 h from printing shear stress; Minimal mortality observed by day 7	—	[156]
		Gelatine	NIH 3T3 fibroblasts	Mechanical blending; Complex coacervation process	After 7 days, decreased cell circularity indicated spreading; Prolonged crosslinking (0–2 h) before printing sharply reduced viability	—	[128, 175]
		Alginate, GelMA, Alginate‐GelMA	GFP + MDA‐MB‐231 cells	Air‐assisted coaxial device	High viability (>90%) and proliferation of MDA‐MB‐231 and fibroblasts over 7 days; GelMA microgels supported attachment, growth, and efficient encapsulation	—	[167]
Wound healing and regenerative medicine		HAMA‐PBA/GelMA	L929 cells	Microfluidics	High proliferation rate after 7 days of culture; Good cell infiltration into bioink; Good tissue adhesion; No cytotoxicity	93% wound closure after 2 weeks in a full‐thickness skin defect on a male rat	[132]
		GelMA/AlgOx	HUVECs	Extrusion fragmentation	HUVECs showed 87% viability after centrifuge‐driven fabrication of AlgOx microgels; cells were distributed evenly throughout printed samples regardless of void fraction via release from sacrificial microgels	—	[130]
		Collagen fibrils	HM/LAP	Mechanical fragmentation	High biocompatibility and biodegradability, promoted fibroblast migration of L929, HUVECs, and hMSCs; No cytotoxicity on the hMSCs viability; Excellent hemocompatibility (hemolysis values <5%)	Excellent ability to promote wound healing by accelerating the blood vessel formation in a full‐thickness skin defect model; Good biodegradability in vivo	[134]
	Bioactive	PEG	hMSCs	Electrospraying	Live/Dead staining showed hMSC viability >88% (1 h), 80% (1 day), and >90% (5–10 days); 3T3 cells maintained >95% viability 30 min post‐injection	—	[127]
Drug delivery	Bioactive	Aldehyde‐modified HA/PNAM	Doxorubicin/3T3 cells/RGD peptides	Photo‐crosslinking and extrusion fragmentation	Good self‐healing ability; pH‐ and redox‐responsive doxorubicin release; Improved 3T3 cells viability (>95%) 30 min after injection	—	[176]
		Alginate	Polyelectrolytes	Microfluidics, polyelectrolyte layer‐by‐layer coating	Low cytotoxicity; High loading efficiency (∼83%) of lipoplexes carrying pEGFP DNA	—	[177]
3D cell culture	Simple	qDMAEMA, MA	—	Precipitation polymerization	Short‐term cell viability >90% after 24 h in anionic and zwitterionic microgels; ATP production in the cells remains unmodified	—	[117]
		GelMA	—	Emulsion photopolymerization	GelMA microgel size influences print fidelity; Higher cell activity in microgels compared to bulk hydrogels	—	[144]
		pNIPAM	—	Precipitation polymerization	3D printed structures for modulating cells with features as small as 50 µm	—	[148]
	Bioactive	GelMA	NIH 3T3 fibroblast cells	Ink‐jet 3D printing	High cell viability (>90%) after printing, spreading of encapsulated cells, and 3D network formation up to 7 days; Mechanical stimulation of encapsulated cells for mechanobiological screening	—	[178]
		Gelatine/GelMA	MSCs and pancreatic β‐cells	Microfluidics	Excellent cell viability, enhanced β‐cell insulin secretion, supportive microenvironment for MSCs	—	[179]
Advanced biofabrication	Simple	NorHA	—	Emulsion photopolymerization	Increased strength and fracture work; Modulus of elasticity is ∼4 times higher than bulk	—	[142]
		PAK, 4‐arm PEG, 4‐arm PEG‐acrylate, chitosan/PVA	—	Mechanical grinding	Adequate adhesion and spreading of fibroblasts seeded on the scaffold surfaces for 4 days	—	[140]
		Acrylamide, MA, PEGDA	—	Precipitation polymerization	NIH 3T3 fibroblasts infiltrated and spread within printed regions after 24 h	—	[180]
		Agarose methacrylate	—	Atomization (Spray‐drying)	Cell viability increased over time; Microgels showed higher viability than bulk hydrogel after 5 days	—	[134]
		pNIPAM	—	Surfactant‐free dispersion copolymerization	Glioblastoma cell line increases cellular elongation from 60% to almost 90%, from day 5 to day 10	—	[126]
		Vinyl‐functionalized gelatine, AA	—	Emulsion polymerization	Accelerated migration and proliferation of fibroblasts and keratinocytes under electrical stimulation conditions at day 7	—	[98]
	Bioactive	Gelatine	Ureolytic bacteria, Sporosarcina pasteurii	Emulsification	Light‐weight inorganic porous structures mimicking natural materials (e.g., trabecular bone) with excellent mechanical strength, bearing up to 3.5 MPa	—	[129]
		Alginates/polyoxazolines/low and high MW GelPa	Yeast (s. cerevisiae), HEK293T cells NIHT3T cells, BJ1‐TERT cells	Flow‐focusing microfluidics	Capability to encapsulate and print microgel yeast cells, mouse embryonic fibroblasts, human fibroblasts, and human embryonic kidney cells; Cell survival and proliferation during encapsulation and extrusion processes from the first day	—	[139]
	Core–shell	CMC/Gelatine‐GelMA	A549, HEK 293 T cells, yeast S. cerevisiae, C. vulgaris, B. subtilis	Flow‐focusing microfluidics	High HEK 293T viability after 6 days; Microgel size minimally affected yeast fermentation efficiency, highlighting their use as whole‐cell biocatalysts	—	[138]
Soft robotics and actuators	Simple	PAMPS	—	Emulsification	Compression‐responsive, color‐changing 3D printed structures with adjustable mechanics and ten‐fold higher fracture toughness	—	[124]
		Chitosan/synthetic polymeric matrices	—	Emulsion polymerization	High tensile strength (0.4–1.0 MPa), strong toughness, and large deformability (elongation at break of 47%–626%)	—	[136]
		Agarose	—	Emulsion polymerization	Degradation in simulated physiological buffer over 1 week, indicating transient stability for biodegradable soft electronics	—	[108]
		Alginate/chitosan	—	Sprayed into a coagulating bath	The inclusion of microgels leads to hydrogels with 20 times higher mechanical strength and hardness compared to single‐network controls.	—	[181]

*Abbreviations*: AA—acrylic acid; AlgOx—oxidized alginate; BSA—bovine serum albumin; CMC—carboxymethylcellulose; ECM—extracellular matrix; GelMA—methacrylated gelatine; GelPa—pentenoic acid modified gelatine; GFP—green fluorescent protein; HA—hyaluronic acid; HAMA‐PBA—hyaluronic acid modified with methacrylate and phenylboric acid groups; hASCs—human adipose stem cells; hiPSCs—human‐induced pluripotent stem cells; HM—methylacrylyl hydroxypropyl chitosan; hMSCs—human mesenchymal stem cells; HUVECs—human umbilical vein endothelial cells; LAP—laponite; MA—methacrylic acid; NRVMs—neonatal rat ventricular cardiomyocytes; OMA—oxidized methacrylated alginate; PAK—Poly(L‐lysine‐ran‐L‐alanine); PEGDA—poly(ethylene glycol diacrylate); PEG—poly(ethylene glycol); PL—human platelet lysates; PNAM—poly(N‐isopropyl acrylamide‐co‐acrylamide‐co‐2‐mercaptoethylacrylamide; pNIPAM—poly(N‐isopropylacrylamide); PVA—poly(vinyl alcohol); SilMA—methacrylated silk fibroin.

### 6.1 Biomedical Potential of Microgel‐Based 3D Constructs for Tissue Engineering

Microgel‐based 3D constructs have emerged as promising and versatile platforms for tissue engineering owing to their versatility in physicochemical properties, modularity, and ability to mimic the native extracellular matrix (ECM). Since 3D printed constructs represent one of the key pillars in tissue engineering, strategies to improve their structural support, mechanical properties, and biological performance are considered crucial for effective tissue regeneration. Beyond biocompatibility, the unique morphological and rheological features of microgel‐based 3D constructs enable improved nutrient diffusion, controlled cell organization, and dynamic microenvironmental tuning for optimal cell development, while allowing precise spatial control through advanced biofabrication strategies [32].

In this context, microgel‐based 3D constructs have been extensively investigated for the engineering of a broad spectrum of tissue engineering applications [116, 121, 133, 141, 156], registering promising outcomes both in vitro and in vivo while mitigating the limitations associated with their bulk counterparts.

**FIGURE 6:**
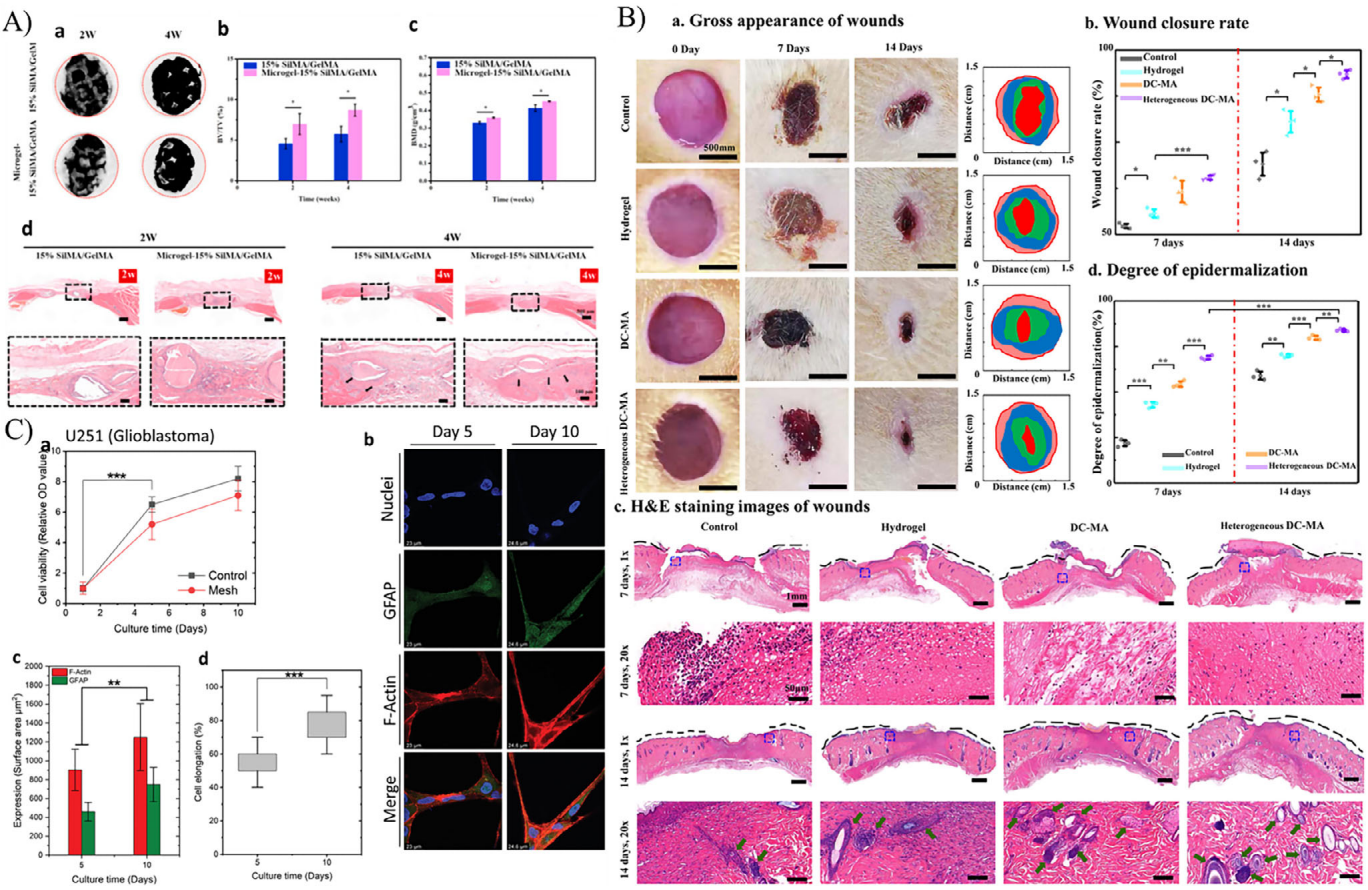
Representative in vitro and in vivo observations showing the diversity of biological contexts in which microgel‐based 3D constructs have been evaluated. These examples highlight that biological performances arise from the interplay of numerous parameters, including microgel design, rheological properties (e.g., jamming behavior), and processing conditions, rather than from application alone. (A) Bone defect model following the implantation of microgel‐based 3D printed constructs. (a) 3D reconstructed micro‐CT images of rat cranial bone defects after 2 and 4 weeks of implantation, (b) BV/TV and (c) BMD analysis at 2 and 4 weeks, (d) H&E‐stained histological sections at 2 and 4 weeks. *p*< 0.05; black arrows indicate newly formed bone. Reproduced (Adapted) with permission from [137]. Copyright 2021, Elsevier. (B) Wound healing model using heterogeneous DC‐MA layers: (a) Gross images of wounds treated with saline, hydrogel, DC‐MA, and heterogeneous DC‐MA at 0, 7, and 14 days; (b) Wound closure rates after 7 and 14 days; (c) H&E staining at 7 and 14 days; (d) Degree of epidermalization at 7 and 14 days. Reproduced (Adapted) with permission from [132]. Copyright 2022 American Chemical Society. (C) Cell culture studies using printed microgel meshes: (a) Viability and cytotoxicity of U251 glioblastoma cells; (b) Confocal images showing F‐actin (red) and GFAP (green); (c) Expression levels of F‐actin and GFAP; (d)Quantification of cellular elongation. Reproduced (Adapted) with permission from [126]. Copyright 2022, JohnWiley & Sons, Inc.

***Bone and cartilage tissue engineering*** represents one of the most extensively explored areas of regenerative medicine, since bone and cartilage frequently suffer from trauma and degenerative diseases, requiring a strong demand for advanced strategies capable of restoring their structural integrity and functionality. Among emerging approaches, microgel‐based 3D matters have demonstrated clear advantages over conventional bulk hydrogels owing to their modularity and intrinsic microporosity, enabling them to provide both mechanical support and an optimal microenvironment for cell differentiation and proliferation. For instance, Chai et al. [137] demonstrated that microfluidic microgels can function as advanced bioink building blocks for bone tissue engineering, by developing cell‐laden microgels featuring a type I collagen core and an alginate shell via a one‐step microfluidic process and then blending with methacrylated silk fibroin (SilMA) and GelMA to enable 3D bioprinting. Acting as a protective environment for cells by protecting them from shear stress during printing, and maintaining nutrient diffusion, allowing cell‐cell communication as well as mimicking the ECM, incorporated microgels within SilMA/GelMA presented higher cell viability, proliferation, and improved osteogenic activity in vitro as compared to the bulk counterpart. Furthermore, the in vivo studies highlighted that microgel‐based scaffolds significantly enhanced bone regeneration outcomes with noticeably reduced rat cranial bone defects observed after 2 and 4 weeks (Figure 6A). Aiming to tackle the poor nutrient diffusion and limited cell‐cell interaction associated with the lack of microporosity of conventional hydrogel bioinks, Flegeau et al. [133] exploited the high porosity and modularity of enzymatically crosslinked hyaluronic acid (HA) microgel bioinks in the formation of mature cartilage in vivo. Beyond the biomimetic cartilage microenvironment provided by the intrinsic characteristics of HA, the shear‐thinning behavior of microgel bioinks protects cells during extrusion, while the specific structural features of porous microgels create interconnected voids between particles, generating a macroporous scaffold after printing. This architecture enhanced cellular activity by improving nutrient diffusion, cell interactions, and cartilage matrix deposition, ultimately promoting chondrogenic tissue formation and maturation in extrusion‐bioprinted constructs after 6 weeks of in vivo implantation. Further, Jeon and team [121] developed a scaffold‐free bioprinting strategy where human stem cells‐only bioink is printed in a photocrosslinkable OMA microgel supporting bath. The contribution of the microgel bath consists initially in supporting nozzle movement, by acting as a fluid during printing, and subsequently provides mechanical support to maintain the structural integrity of the printed construct. The approach is considered a paradigm shift in scaffold‐free bioprinting, allowing the fabrication of complex 3D tissue constructs with high cell density and precise architecture, while successfully supporting the differentiation into bone and cartilage tissues. It holds great potential for application in regenerative medicine, drug screening, and progressive biology.

***Cardiovascular tissue engineering*** is another field of tissue engineering that has experienced rapid progress in recent years following extensive experimental research. However, the fabrication of stable, hollow, cylindrical, and vertically extended vascular structures using conventional 3D printing techniques remains one of the main challenges that needs to be addressed. In this context, Jin et al. [115] developed a “printing‐then‐gelation” strategy, in which a gelatin–alginate bioink was extruded into a granular Carbopol support matrix, followed by dual crosslinking through thermal and ionic mechanisms. This approach enabled the fabrication of hydrogel and vascular‐like tubular constructs using NIH 3T3 fibroblast‐laden bioinks, maintaining over 80% cell viability after three days, demonstrating the potential of this method for vascular scaffold fabrication. Outside vascular structures, the bioengineering of cardiac tissues represents another major challenge within cardiovascular tissue engineering. For example, starting from a simple matrix, Fang et al. [141] demonstrated that a microgel‐based biphasic bioink can function both as a high‐performance printing material and as a supportive medium for 3D bioprinting of cardiac constructs. The authors used a gelatin bioink encapsulating human‐induced pluripotent stem cells (hiPSCs) to fabricate cardiac tissues and organoids, supporting robust stem cell proliferation and the ability to differentiate. The contribution of microgels in providing structural support, improving printing performance, and assuring a cytoprotective environment was demonstrated, since cardiac differentiation was successfully induced, resulting in the formation of beating cardiac organoids within eight days. Furthermore, by combining the microgel‐based bioink within a sequential printing in a reversible ink template (SPIRIT) strategy, the authors were able to fabricate a ventricle model containing a perfusable vascular network, demonstrating the potential of this approach for engineering complex cardiovascular tissues.

Besides, microgel‐based biofabrication strategies were also used in the ***engineering of solid organs***, which continue to represent a major challenge in biomedicine owing to their structural complexity, hierarchical organization, and strongly regulated multicellular functionality. In this respect, Subramaniam et al. [116] presented an accurate and precise approach for 3D bioprinting functional human liver tissue models by formulating cell‐laden discoids composed of collagen‐1, embedded in a highly permeable support medium of packed polyethylene glycol (PEG) microgels. The construct presented self‐organization, cell cohesion, and expression of key liver markers, as proven by histologic and immunohistochemical tests, whereas it maintained stable urea and albumin production for three weeks and expressed over 100 genes associated with absorption, distribution, metabolism, and excretion, closely resembling native liver tissue. Furthermore, the constructs also demonstrated active metabolic functionality through enzymatic activity, highlighting their potential for pharmacological and toxicological applications. Then, to facilitate the fabrication of functional biological constructs with biomimetic structural organizations and high resolution through embedded 3D bioprinting, Zhang et al. [120] developed a submicron cell‐friendly cationic‐crosslinked k‐carrageenan microgel system with excellent shear‐thinning and self‐healing properties. The submicron microgel support medium allowed the successful bioprinting of bone marrow mesenchymal stem cell (BMSC)‐laden complex 3D tissue and organ‐like structures with fine architectural features and high shape fidelity, while exhibiting high cell viability (>92.1%) along with robust cellular growth.

In addition, owing to the structural and modular advantages over conventional bulk hydrogels, microgel‐based 3D printed constructs are gaining increasing attention across ***diverse areas of tissue engineering***, offering new opportunities in the development of more sophisticated and functional tissue constructs [8, 23, 128]. In the light of this, jammed microgel inks allowing the direct 3D printing of cell‐laden constructs without additional support materials, along with microgel‐based scaffolds with tunable void fraction capable of enhancing mass transport, cell infiltration, and tissue integration, have been widely explored [8, 23]. In a comprehensive study, Song et al. [175] demonstrated that transglutaminase‐mediated crosslinking enables precise tuning of the rheological properties and printability of gelatin microgel–gelatin composite bioinks for extrusion‐based 3D bioprinting. Albeit the structural fidelity of the construct was significantly improved, the authors underlined the need to identify an optimal balance between printability and structural stability to preserve cell viability while maintaining high printing fidelity. Further, Pal et al. [167] paved the way for developing a scalable strategy for the fabrication of cell‐laden GelMA microgels, demonstrating high viability and proliferation of human dermal fibroblasts following 3D bioprinting into complex structures. The resulting constructs exhibited homogeneous cell distribution, sustained metabolic activity, and structural versatility, highlighting their potential for connective tissue engineering applications. Building upon earlier jammed microgel systems, recent approaches have introduced intrinsically bioactive granular bioinks, such as blood‐derived protein microgels, which combine nozzle‐induced jamming with cell‐instructive properties to further enhance both printability and biological functionality. In this context, very recently Ribeiro et al. [156] engineered granular bioinks derived from blood proteins that exploit nozzle‐induced jamming to fabricate cell‐instructive 3D architectures. The formulated systems presented high shape fidelity as well as exhibited intrinsic biocompatibility and biomimicry, owing to the inherent bioactivity of the protein‐based microgels.

### 6.2 Biomedical Potential of Microgel‐Based 3D Constructs in Wound Healing and Regenerative Medicine

Wound healing is a highly dynamic and complex process, involving multiple stages characterized by specific cellular and physiological responses, each requiring specific therapeutic interventions [182]. Any aberrant alterations in this process can lead to impaired healing, infection, or chronic wounds. Besides physical and psychological burden, wound healing disorders significantly impact quality of life, reduce productivity, and impose substantial healthcare costs [183]. Wound healing remains a major global healthcare challenge, driving the need for advanced, non‐invasive therapeutic strategies within the broader framework of regenerative medicine, aiming to combine mechanical stability and flexibility with high biofunctionality and dynamic interaction with the wound microenvironment. In this regard, microgel‐based 3D systems offer promising potential by allowing dynamic adaptation to the evolving wound microenvironment and providing stage‐specific therapeutic responses. For instance, Feng et al. [132] proposed a dynamic crosslinked microgel assembly (DC‐MA) bioink that promotes favorable biological responses by combining high microporosity and intrinsic bioactivity. The system, based on HAMA‐PBA and GelMA microgels assembled via a dopamine‐modified hyaluronic acid crosslinker, supported high viability of encapsulated L929 cells following extrusion, as well as enhanced cell infiltration and proliferation owing to its porous architecture and large surface area. These biological advantages translated into significant in vivo performance, achieving approximately 93% wound closure after 2 weeks, highlighting its strong potential for tissue engineering and regenerative medicine applications, particularly in wound healing (Figure 6B). Similarly, Li et al. [135] developed a biomimetic, collagen fibril‐like injectable hydrogel via in situ photo‐crosslinking of self‐assembled methylacrylyl hydroxypropyl chitosan‐laponite (HML) nanoparticles. The formulated microgels combined low stiffness, high compressive strength, and sustained drug release with high‐fidelity 3D printability. Further, the authors proved the high biocompatibility and biodegradability along with excellent ability to promote wound healing by accelerating the blood vessel formation during wound healing in a full‐thickness skin defect model in vivo. Recent work has introduced 3D printed bilayer adipose–skin constructs for advanced in vitro tissue modeling, embedding NIH 3T3 spheroids in GelMA microgels within a PCL scaffold for the adipose layer, and GelMA with fibroblasts and GFP‐HUVECs forming the skin layer [184]. The proposed concept registered enhanced adipogenic differentiation, gene expression, and vascularization compared to conventional 2D systems, attributed to the high microporosity and tunable mechanical properties of microgels, which promote nutrient diffusion, vascularization, and regulate cell behavior. Moreover, the hydrated, biomimetic microenvironment of GelMA microgels promotes cell–cell and cell–matrix interactions, supporting effective adipose–skin integration and the formation of complex tissue interfaces. Overall, this strategy assures precise spatial organization of multiple cell types, highlighting the potential of 3D microgel surfaces to replicate tissue interfaces.

### 6.3 Biomedical Potential of Microgel‐Based 3D Constructs in Drug Delivery

Beyond their contribution in tissue engineering and regenerative medicine, microgel‐based 3D constructs have arisen as versatile and amenable platforms for drug delivery, as their modular structure and tunable properties allow efficient and controlled encapsulation along with spatiotemporal release of therapeutic agents (e.g., small biomolecules, proteins, and nucleic acids) [61]. Additionally, their microscale polymeric network structure, crosslinking density, and dynamic responsiveness make them particularly suited not only for programmable drug delivery but also customization for specific therapeutic functions [63]. Recently, research demonstrated that dynamically crosslinked thermoresponsive granular hydrogels can also function as effective drug delivery systems for cancer therapy. In this context, a thiol‐functionalized thermo‐responsive polymer incorporated into oxidized hyaluronic acid microgel assemblies, allowing the covalent loading of doxorubicin. The resulting construct presented a pH‐ and redox‐responsive controlled release of cargo, leading to a significant reduction in the viability of MCF‐7 cancer cells [176]. In another work, Carvalho et al. [177] employed layer‐by‐layer (LbL) constructed biomimetic microgels for efficient localized gene delivery by embedding lipoplexes containing plasmid DNA within polyelectrolyte alginate‐based microgels. The proposed concept exhibited low cytotoxicity, supporting favorable cell‐material interactions. In addition, it demonstrated a high loading capacity of lipoplexes (∼83%) and promoted surface‐mediated in vitro transfection of MCF‐7 cells, providing a new suitable route for cell adhesion and local gene delivery.

### 6.4 Biomedical Potential of Microgel‐Based 3D Constructs for Advanced 3D Cell Culture

3D cell culture systems provide a more realistic physiological microenvironment by allowing cells to proliferate within spatially defined architectures that promote complex multicellular assembly, dynamic cell–cell and cell–matrix interactions, and the development of tissue‐like organization [185]. In this framework, the particular nature of microgel‐based 3D constructs offers transformative biomedical potential by tackling the main challenges associated with traditional bulky hydrogels, such as poor nutrient diffusion and restricted cell migration. By replicating the essential features of the extracellular matrix at the microscale, they facilitate 3D cell encapsulation, controlled cell‐matrix interactions, and efficient diffusion of nutrients and signaling molecules [186]. For example, Sun et al. [179] developed porous gelatine/GelMA microgels co‐encapsulating pancreatic β‐cells and mesenchymal stem cells (MSCs) able to control hyperglycemia in diabetes. The authors noted that porous structure supported cell growth and proliferation whereas allowing efficient nutrient and waste transport, helping to preserve a functional microenvironment. In addition to encapsulation, microgels constitute substrates for cell growth on their surfaces. Their high surface‐to‐volume ratio compared with conventional monolayer cultures allows the expansion of more cells within a reduced medium volume, suggesting the potential as platform for clinical β cell transplantation. It was also demonstrated that jammed polyelectrolyte microgels can influence cellular responses in a composition‐dependent manner, with short‐term viability governed by their chemical nature [117], while ultrasonic microplotting can generate high‐resolution patterning of microgel bioinks, allowing precise spatial organization of microgel‐based constructs beyond the limitations of conventional layer‐by‐layer techniques [148]. Further, aiming to address the limitations of existing strategies for investigating cellular responses to mechanical stimulation, Sakthivel et al. [178] developed a stretchable 3D microgel‐based cellular microarray platform for high‐throughput screening of cell mechanical responses, that allows to study the mechanotransduction phenomena in physiologically relevant environments. Owing to the intrinsic microporosity and tunable mechanical properties of the microgel system, the platform supported high cell viability and the formation of organized 3D cellular structures. Moreover, the applied mechanical stimulation induced significant changes in cell morphology, alignment, and cytoskeletal organization. These responses highlight the ability of the proposed concept to recapitulate dynamic cell–matrix interactions and provide valuable insights into how mechanical cues regulate cellular behavior.

### 6.5 Biomedical Potential of Microgel‐Based 3D Constructs in Advanced Biofabrication

Extending their role as supportive platforms in 3D cell culture, microgel‐based 3D printed constructs have further emerged as promising approaches for advanced biofabrication, owing to their ability to integrate structural precision with biomimetic functionality. Their tunable mechanical properties, high hydration, and adaptable chemistry allow the formation of dynamic, cell‐supportive microenvironments. At the same time, their modular nature allows assembly into complex, porous architectures with controlled cellular organization and enhanced mass transport, supporting the development of sophisticated tissue constructs and physiologically relevant in vitro models [187]. To advance practical tissue engineering, Reineke and colleagues [139] developed an on‐chip microfluidic platform for continuous production of alginate/poly(oxazoline)/GelPa microgels encapsulating various cell types (yeast, HEK293T, NIH3T3, BJ1‐TERT). These microgels were photo‐crosslinked in‐flow and directly printed into perfusable 3D scaffolds, addressing scalability and post‐processing challenges. In another work, Hirsch et al. [129] developed bacteria‐laden gelatine microgels containing ureolytic *Sporosarcina pasteurii* bacterial strains, enabling the 3D printing of mechanically resilient organic–inorganic composites through microbially‐induced calcium carbonate mineralization. The resulting biocomposite materials achieved mineral contents of up to 93 wt.% and bore loads up to 3.5 MPa, having the potential to be used in repairing damaged or partially degraded mineral‐based materials, including bone structures. Polysaccharide‐derived microgels also represent a major class of natural formulations used in advanced biomedical applications. Mukundan et al. [134] produced agarose methacrylate microgels through spray drying and printed grid‐like scaffolds with good cell responsiveness, supporting MSCs proliferation, as evidenced by increasing DAPI‐stained nuclei counts over time. Their microporous architecture enhanced nutrient and oxygen diffusion, resulting in significantly higher cell survival by day 5 compared to bulk hydrogels. These microgels highlight strong potential for biofunctional printing, offering tunable architecture, mechanical robustness, and excellent biocompatibility. In a recent work, Sayed et al. [126] reported the development of thermo‐sensitive PNIPAM‐based jammed microgel inks stabilized by terpyridine–Fe^2^^+^ coordination, enabling reversible stiffness tuning. Glioblastoma cells cultured on 3D‐printed meshes showed increasing viability over 10 days, with elevated F‐actin and GFAP expression indicating cytoskeletal remodeling and active proliferation. Cellular elongation rose from 60% to nearly 90% between days 5 and 10, confirming high cytocompatibility and demonstrating the potential of these constructs for neural tissue modeling (Figure 6C).

### 6.6 Biomedical Potential of Microgel‐Based 3D Constructs in Soft Robotics and Actuators

At the interface of materials science and bioinspired engineering, microgel‐based 3D constructs are redefining soft robotics and actuator design by introducing materials that behave more like living tissues than conventional solids [188, 189]. Their unique mechanical characteristics endow them with tissue‐like compliance, while their shear‐thinning and self‐healing properties enable the precise fabrication of complex, deformable architectures. Moreover, the programmable composition and crosslinking of microgels allow dynamic, stimuli‐responsive actuation—mimicking biological processes such as swelling, contraction, and movement. Their porous and modular nature further supports multifunctionality, such as sensing and biointeractive capabilities within a single platform [190]. For example, Hirsch et al. [124] proposed the strategy of combining a microgel network with a covalently crosslinked polyacrylamide‐based matrix to improve mechanical performance. The resulting double‐network microgels exhibited increased strength, toughness, and stretchability, at the same time maintaining good printability and structural stability, making them suitable for soft robotics applications, such as soft actuators and deformable structures capable of bearing mechanical loads. In another work, Huo et al. [108] reported the development of 3D printed ionic microgels inserted in a soft polymer matrix to create mechanically robust, conductive junctions. The agarose microgels were fabricated via controlled polymerization and printed using extrusion‐based techniques to form structured ionic pathways. The resulting materials combine toughness, flexibility, and stable ionic performance, showing strong potential for stretchable sensors and soft actuators applications.

Collectively, these studies underline the transformative potential of microgel‐based 3D printed constructs across diverse biomedical applications, owing to their ability to integrate mechanical integrity, cellular support, and therapeutic functionality within biomimetic systems that recapitulate native tissue dynamics. Moving forward, bridging the gap between experimental success and clinical implementation remains a critical challenge, highlighting the careful consideration of key practical, translational, and regulatory aspects.

## 7 Perspectives in Biomedicine and Considerations for Clinical Translation

Advances in 3D bioprinting and bio‐fabrication have enabled the creation of increasingly complex and functional tissue constructs, opening new pathways to bridge the gap between laboratory‐scale innovation and clinical translation. Among the most promising developments is the integration of microgel‐based systems and in situ bioprinting approaches, both of which address key challenges related to structural integrity, biological fidelity, and translational scalability [191, 192].

Furthermore, by merging with advanced imaging and computational modeling, these approaches are evolving toward truly patient‐specific biomanufacturing. Real‐time in situ bioprinting guided by medical imaging can replicate complex anatomical architectures with remarkable submillimeter precision [191, 192], marking a pivotal step toward personalized, high‐fidelity tissue restoration and functional regeneration. Complementary innovations in microparticulate bioinks have further expanded the scope of printable materials, supporting the fabrication of constructs with enhanced porosity, gradient architecture, and mechanical heterogeneity [7, 21]. Collectively, these advances demonstrate how microgel‐based and in situ bioprinting technologies are pushing the boundaries of precision biofabrication, shifting from static, conventional hydrogel‐based scaffolds toward dynamic, living constructs that better mimic the complexity of native tissues [7, 13, 24, 26]. Although biomedical applications of microgel‐based 3D constructs are expanding rapidly across multiple domains, including bone, cartilage, vascular, and soft tissue engineering, the translation of these materials into the clinic remains hindered not only by general regulatory barriers but also by scientific and technical challenges inherent to their granular and jamming‐dependent material behavior [193]. The same modularity, versatility, structural, and biological adaptability in constructing tissue‐like architectures, difficult to obtain with bulk hydrogels, bring additional complexity in process control, reproducibility, and safe assessment.

Batch‐to‐batch formulation and reproducibility represent primary technical challenges, since microgels represent a population of colloidal particles, often with heterogeneous internal structure and anisometric shape as well as specific size distribution, crosslinking density, and surface chemistry, whereas minor variations of these features can significantly alter packing behavior, yield stress, and, respectively, printability [192, 193]. As a result, batch‐to‐batch variability leads to constructs with different architectural and cellular microenvironments, complicating standardization under Good Manufacturing Practice (GMP) requirements.

**Box 3 |Practical and regulatory considerations of microgel‐based 3D printed scaffolds**

Practical Challenges

Formulation reproducibility: Variations in particle size and packing affect resolution and mechanical stability.

Process‐dependent parameter behavior: Printing parameters strongly influence jamming, filament formation, and final construct properties.

Post‐printing stabilization: Requires secondary crosslinking; may cause heterogeneous bonding and cytotoxicity.

In vivo performances: Porosity and modular degradation complicate the prediction of vascularization and tissue integration.

Regulatory Challenges

Standardization: No universal standards for safety, efficacy, or quality evaluation.

Product Classification: Unclear regulatory pathways (medical device, combination product, or ATMP).

GMP Compliance: Meeting Good Manufacturing Practice requirements for clinical use.

Clinical Trials: Designing studies addressing safety, ethics, and long‐term monitoring.

Ethical & Data Considerations: Protecting patient‐derived data and addressing cell source ethics.

In addition, microgel bioinks are characterized by printer‐dependent rheological behavior. Besides polymer chemistry, the mechanical behavior of microgel constructs is strongly impacted by the particle packing fraction and applied shear history [98]. Therefore, the rheological and structural characteristics of the same microgel bioink formulation may be dissimilar when processed under different printing conditions (e.g., extrusion pressure, nozzle diameter, printing speed), determining the variability in filament formation, shape fidelity, and mechanical stability of the printed construct.

Further, post‐printing stabilization of microgel‐based 3D constructs is usually achieved by secondary annealing and interparticle crosslinking, which may introduce additional concerns (e.g., heterogeneous bonding between particles and potential cytotoxicity from photoinitiators or crosslinking agents) that can impact not only the mechanical integrity of the construct but also cell behavior and the reproducibility of biological outcomes [98, 194].

Long‐term in vivo predictability further hinders translation. Owing to their modular structure, the classical degradation kinetics followed by bulk hydrogel are fundamentally altered in these systems. Degradation proceeds at the level of individual particles and at the level of interparticle connections, leading to spatio‐temporally heterogeneous material evolution [99] and complicating assessment and prediction of long‐term in vivo performance and safety [193]. Ensuring long‐term biocompatibility and controlled degradation remains a critical challenge for clinical scalability [195, 196]. Besides, long‐term storage is another technical barrier since the microgel particles are prone to aggregation or changes in packing behavior during storage, complicating commercialization and distribution.

Beyond technical issues, regulatory and logistical considerations must also be tackled. The ethical dimensions of personalized bioprinting (e.g., the use of patient‐derived cells, data privacy, and long‐term monitoring) underscore the need for regulatory harmonization and transparent guidelines to build trust among researchers, clinicians, and patients [193]. It is also important to note that the commercialization of 3D printed constructs faces significant logistical and economic barriers, including high production costs, limited scalability, and reliance on specialized equipment [197]. Therefore, the utmost priorities for translating these constructs from proof‐of‐concept to clinically functional tissues include not only the material innovation but also robust supply chains, stringent quality control, and well‐trained clinicians. Without addressing these critical factors, widespread clinical adoption will remain slow, despite rapid technological advances [198].

Looking forward, advancing microgel‐based 3D printed structures will require the convergence of material science innovations, digital technologies, and regulatory frameworks to ensure safe, reproducible, and cost‐effective clinical translation. Importantly, interdisciplinary collaboration among scientists, engineers, clinicians, and policymakers will be key to bridging the gap between laboratory breakthroughs and real‐world clinical applications. By aligning material innovation with manufacturing and ethical oversight, these technologies hold the promise of moving beyond proof‐of‐concept constructs toward personalized, scalable, and therapeutically effective biomedical solutions that can redefine the future of biomedicine.

## Conflicts of Interest

The authors declare no conflicts of interest.

## Data Availability

Data sharing not applicable to this article as no datasets were generated or analyzed during the current study.
